# Supplementary material for: Mono- and biallelic variant effects on disease at biobank scale
Source: Nature. 2023 Jan 18;613(7944):519–25. doi: 10.1038/s41586-022-05420-7 (PMC9849130; doi:10.1038/s41586-022-05420-7)
Supplement: Supplementary file 1 — This file contains Supplementary Notes, Supplementary Tables 5–7 and 9, Supplementary References and a list of FINNGEN members. [file 41586_2022_5420_MOESM1_ESM.pdf]

nature portfolio

https://doi.org/10.1038/s41586-022-05420-7

Supplementary information

Mono- and biallelic variant effects on disease at biobank scale

In the format provided by the authors and unedited

Supplementary Information

Table of Contents

SUPPLEMENTARY INFORMATION 1

SUPPLEMENTARY FIGURES 2

SUPPLEMENTARY TABLES 3

SUPPLEMENTARY NOTES. 11

REFERENCES 19

FINNGEN MEMBERS 21

Supplementary Figures

Supplementary Figures S1. Separate pdf. Longitudinal survival curves showing disease onset of homozygous, heterozygous and wildtypes of variants with recessive associations in FinnGen.

Supplementary Figures S2. Separate pdf. Longitudinal survival curves showing disease onset of homozygous, heterozygous and wildtypes of known likely pathogenic or conflicting disease variants.

Supplementary Tables

Glossary column names in Supplementary Tables

phenotype, disease endpoint name

variant, chr:pos:ref:alt

chr, chromosome,

pos, position in GRCH38

ref, reference allele

alt, alternate allele

rsid, rsid

INFO, overall genotype imputation INFO score

AF, allele frequency in FinnGen data

AN, total allele number in FinnGen data

AC_Hom, allele count in individuals homozygous for the alternate allele in FinnGen

enrichment_nfsee, AF in Finns/ AF in NFSEE (Non-Finnish-non-Swedish-non-Estonian)

grch37_locus, position in GRCH37

cs, included in credible set after fine-mapping (additive model, method: SuSiE)

pip, posterior inclusion probability after fine-mapping (method: SuSiE)

add_pval, SAIGE p-value (additive model)

rec_pval, SAIGE p-value (recessive model)

beta, SAIGE beta (log OR scale)

sebeta, SAIGE beta standard error

top_associated_phenotype_add, most significantly associated phenotype (additive model)

top_associated_phenotype_rec, most significantly associated phenotype (recessive model)

top_add_pval, p-value of most significant association (additive model)

top_rec_pval, p-value of most significant association (recessive model)

variant_category, gnomAD variant category

gene, gene from VEP (Variant Effect Predictor) annotation to which most severe consequence applies

R6, FinnGen data release 6 (n=234,553)

R4, FinnGen data release 4 (n=176,899))

ClinVar: Category, category grouped according to 'ClinVar_ReviewStatus'

ClinVar: Review, 'ClinVar_ReviewStatus' as given originally in ClinVar
Mode of inheritance (OMIM), Mode of inheritance annotated from OMIM for this gene ('both': both dominant and recessive inheritance modes annotated)

Supplementary Table S1. Any coding variant association that has a p-value < 10^{-7} in the additive model or a p-value < 10^{-7} in the recessive model (all available disease endpoints).

Supplementary Table S2. (A more detailed version of Table 1.) Recessive associations. Only the most significant variant is shown per locus. mis, missense; pLoF, probable loss-of-function; hom, homozygotes in FinnGen; R6, FinnGen data release 6 (n=234,553); UKBB, UK biobank (n=420,531).

Supplementary Table S3. Summary statistics of coding variants with any recessive association, i.e. recessive p-value < 10^{-7} and recessive p-value two orders of magnitude below additive p-value (all available disease endpoints, associations with p-value < 0.01).

Supplementary Table S4. Simulation results of genotype and phenotype data to assess the output of our recessive and additive model in the presence of a strictly recessive or additive association.

Supplementary Table S5. Variants with recessive associations in FinnGen that overlap known GWAS hits. Adjacent variants with r^2>0.25 associated with the same trait or same parent trait are considered as one locus and only the most significant variant shown per locus. The first five columns show gene, rsid, associated phenotype, recessive p-value and beta in FinnGen. Columns six to eight give the known GWAS phenotypes, SNPs and p-values. LD (r2>=0.1) of the known GWAS SNPs to respective SNPs in FinnGen is given in the last column. FinnGen, FG.

The six large homozygous effects in Supplementary Table 3 that we thus better captured with a recessive GWAS model have been previously mentioned for two out of the six variants: Homozygosity of rs601338 in *FUT2* has been shown to protect from intestinal infection[1]. Homozygosity of rs689 in *INS* had a significantly stronger effect on Type 1 Diabetes than in heterozygotes[2] and was previously associated with age-at-diagnosis of Type 1 Diabetes (P-value = 0.001), with homozygosity for the protective T allele delaying the onset of Type 1 Diabetes by approximately 2 years in these families[3]. (In our results, the direction of effect is the same, however the reference and alternative allele are swapped, see also Table 2 in the manuscript.)

Gene	rsid	Lead Phenotype FG	p-value FG	beta FG	known GWAS … phenotype in LD	... SNP	… r2	… p-value
UGT1A6	rs1105879	Cholelithiasis	4.10E-11	0.09	Gallstone disease; Serum bilirubin levels in metabolic syndrome	rs2070959; rs2070959	0.8; 0.8	$9x10^{-11}$; $2x10^{-14}$
IGHG3	rs74093865	Immunodeficiency w. predominantly antibody defects	4.40E-08	0.5	Composite immunoglobulin trait (IgG/IgM)	rs74093831	0.72	$8x10^{-9}$
FUT2	rs601338	Intestinal infectious diseases	4.00E-09	-0.07	Number of common colds;Vitamin B12 levels	rs601338; rs492602	1; 0.99	$4x10^{-7}$; $5x10^{-17}$
TMEM214	rs1124649	Pain (limb, back, neck, head abdominally)	3.80E-11	-0.06	CRP level; polymyalgia rheumatica	rs11681145	0.78;1	$9x10^{-12}$; 0.004
INS	rs3842753	Type 1 diabetes	1.20E-41	0.24	Type 1 diabetes	rs689	0.99	$5x10^{-196}$
SLCO1A2	rs10841795	Cardiac murmurs and other cardiac sounds	1.40E-08	1	Blood metabolite levels	rs12829704	0.6	$9x10^{-21}$

Supplementary Table S6 Variants with recessive associations in FinnGen that could be tested for replication in the UK biobank[4]. The last three rows colored in brown/grey show recessive associations of different coding variants in the UK biobank than the coding variant with a genomewide significant hit in FinnGen. UK biobank, UKB; FinnGen, FG; MAF, minor allele frequency.

Gene	rsid	Phenotype FinnGen	Phenotype UKBB	MAF FG	MAF NFE gnomAD	p-value FG	beta FG	p-value UKB	beta UKB
APPL2	rs138491961	noninflammatory disorders of uterus, except cervix	N85 noninflammatory disorders of uterus, except cervix	0.0032	0.0015	3.69E-08	5.54	NA	NA
CEP250	rs56259282	Varicose veins	I83 Varicose veins of lower extremities	0.0046	0.012	1.27E-08	4.33	0.027	1.97
C10orf90	rs139123090	Conductive and sensorineural hearing loss	H90 Conductive and sensorineural hearing loss	0.0085	0.0045	2.25E-12	5.96	0.00012	56.7
PCK1	rs201186470	Glucose regulation and pancreatic secretion	E16 Other disorders of pancreatic internal secretion	0.011	0.00045	4.65E-08	9.77	NA	NA
GJB2	rs398123814	Sensorineural hearing loss	H90 Conductive and sensorineural hearing loss	0.012	0.0092	2.86E-28	6.49	3.11E-08	82.0
C15orf40	rs115868366	Demyelenating diseases of the central nervous system	Multiple sclerosis	0.013	0.018	3.94E-08	11.3	0.058	2.53
CASP7	rs141266925	Other cataract	H26 Other cataract	0.015	0.0035	2.47E-16	4.77	NA	NA
EBAG9	rs1422	Female infertility	N97 Female infertility	0.056	0.041	1.60E-11	2.0	0.0018	3.90
SERPINA1	rs28929474	Emphysema	J43 Emphysema	0.02	0.018	2.16E-21	28.0	1.43E-26	20.6
INS	rs3842753	Type 1 diabetes	Type 1 diabetes	0.79	0.72	5.71E-46	0.28	6.31E-23	0.35
IGHG3	rs74093865	Immunodeficiency w. predominantly antibody defects	D80 Immunodeficiency w. predominantly antibody defects	0.4	0.3	4.44E-08	0.50	0.027	0.53
UGT1A6	rs1105879	Cholelithiasis, broad definition with cholecystitis	Cholelithiasis and cholecystitis	0.45	0.35	1.53E-11	0.08	1.58E-10	0.16
SLCO1A2	rs10841795	Cardiac murmurs and other cardiac sounds	R01 Cardiac murmurs and other cardiac sounds	0.11	0.14	1.38E-08	1.03	0.47	0.14
TMEM214	rs1124649	Pain (limb, back, neck, head abdominally)	General pain for 3+ months	0.28	0.31	3.79E-11	-0.06	0.41	-0.10
ZNF415	rs10410030	Mild mental retardation	F81 Specific developmental disorders of scholastic skills	0.097	0.15	1.56E-08	2.10	0.42	-0.37
FUT2	rs601338	Intestinal infectious diseases	Intestinal infection	0.37	0.48	4.01E-09	-0.07	0.21	-0.03
CASP7	rs2227309	Other cataract	H26 Other cataract	0.28	0.26	NA	NA	0.016	0.07
APPL2	rs2272495	noninflammatory disorders of uterus, except cervix	N85 noninflammatory disorders of uterus, except cervix	0.23	0.20	NA	NA	0.0085	0.18
PCK1	rs1804160	Glucose regulation and pancreatic secretion	E16 Other disorders of pancreatic internal secretion	0.043	0.023	NA	NA	0.038	1.67

Supplementary Table S7. Variants with recessive associations in FinnGen that could be tested for additive associations in the UK biobank (UKBB)[4]. The columns show gene, rsid, phenotype name in UKBB, additive p-value and beta in UKBB and the phenotype name in FinnGen.

Gene	rsid	Phenotype UKBB	p-value	beta	Phenotype FinnGen
ACOXL	rs80265967	Gestational diabetes	>0.05	NA	Diabetes mellitus in pregnancy
APPL2	rs138491961	noninflammatory disorders of uterus, except cervix	>0.05	NA	N85 Other noninflammatory disorders of uterus, except cervix
CASP7	rs141266925	Cataract	4.50E-12	0.7	Cataract
C10orf90	rs139123090	Conductive and sensorineural hearing loss	>0.05	NA	Conductive and sensorineural hearing loss
C15orf40	rs115868366	Demyelenating diseases of the CNS	1.40E-02	0.34	multiple sclerosis
CEP250	rs56259282	Varicose veins	>0.05	NA	Varicose veins
CLCN1	rs55960271	Diseases of the myoneural junction and muscle	>0.05	NA	Myasthenia gravis and other myoneural disorders
CNGB1	rs201162411	Hereditary retinal dystrophy	>0.05	NA	Hereditary retinal dystrophy
DMBT1	rs189221852	Nephropathy	2.20E-04	5.3	Non-proliferative glomerulonephritis
EBAG9	rs1422	Female infertility	>0.05	NA	Female infertility
IGHG3	rs74093865	Immunodeficiency w. predominantly antibody defects	>0.05	NA	Immunodeficiency with predominantly antibody defects
INS	rs3842753	Type 1 diabetes	9.10E-20	0.28	Type 1 diabetes
PCK1	rs1804160	Other disorders of pancreatic internal secretion	>0.05	NA	Glucose regulation and pancreatic secretion
PLTP	rs56126980	Unspecified lump in breast	>0.05	NA	Lump or mass in breast
SERPINA1	rs28929474	Emphysema	2.30E-19	1.3	Emphysema
SLCO1A2	rs10841795	Cardiac murmurs and other cardiac sounds	>0.05	NA	Cardiac murmurs and other cardiac sounds
TMEM214	rs1124649	Pain (limb, back, neck, head abdominally)	4.00E-03	-0.13	polymyalgia rheumatica
UGT1A6	rs200711686	Cholelithiasis	1.40E-06	0.064	Cholelithiasis
USP5	rs200723728	Endometriosis of ovary	>0.05	NA	Endometriosis
USP8	rs147742292	Postpartum haemorrhage	>0.05	NA	Postpartum haemorrhage
ZNF415	rs10410030	Mild mental retardation	2.90E-02	0.31	Mild cognitive impairment

Supplementary Table S8. Most significant disease associations of all 15140 coding variants annotated in ClinVar.

Supplementary Table S9. Likely benign ClinVar variants that are the most likely causative (top PIP) variant in one GWAS locus. Genomic locations are given as rsids and GRCh38 coordinates (chromosome, position in bp, reference and alternate allele, separated by ":"). PIP, posterior inclusion probability (statistical finemapping[5]); AF, allele frequency in FinnGen; AD, autosomal dominant; AR autosomal recessive; KELA, Finnish Social Insurance Institution. All listed variants are missense variants.

Gene	GRCh38	rsid	AF	OMIM phenotype of gene	GWAS phenotype in FinnGen	beta	p-value	PIP
ARMS2	10:122454932:G:T	rs10490924	0.24	Age-related macular degeneration (association)	Age-related mac. degen.	1.06	1.90E-104	0.08
					Other retinal disorders	0.27	1.78E-46	0.08
					Diabetic retinopathy	0.16	3.92E-22	0.08
ATM	11:108272729:C:G	rs1800057	0.01	Ataxia-telangiectasia AR;	Malignant neoplasm	0.28	2.58E-08	0.4
				Breast cancer susceptibility	Leiomyoma of uterus	0.45	1.13E-09	0.22
INS	11:2159830:T:G	rs3842753	0.79	Diabetes m. (neonatal, AR, AD, MODY)	Diabetic maculopathy	0.32	1.04E-10	0.44
					Diabetic retinopathy	0.32	5.55E-19	0.51
HNF1A	12:120978847:A:C	rs1169288	0.37	Diabetes m. (AR, AD MODY)	Statin medication	0.08	1.55E-14	0.86
KRT5	12:52519884:C:T	rs11170164	0.1	Epidermolysis bullosa simplex, AR, AD	Malignant neoplasm of skin	0.21	1.34E-12	0.94
RPL3L	16:1947063:G:A	rs147972626	0.01	Cardiomyopathy, dilated, AR	Atrial fibrillation and flutter	0.45	6.23E-11	0.49
TYK2	19:10352442:G:C	rs34536443	0.03	Immunodeficiency, AR	KELA Reimbursement (Gastrointestinal)	-0.3	6.09E-09	0.7
CFH	1:196690107:C:T	rs1061170	0.56	Complement factor H deficiency, AR, AD; Macular deg. (association)	Disorders of choroid and retina	-0.11	5.18E-16	0.02
					Diabetic retinopathy	-0.09	2.05E-10	0.02
PCSK9	1:55039974:G:T	rs11591147	0.04	Hypercholesterolemia, familial, AD	Hypercholesterolaemia	-0.43	1.33E-16	1
					Coronary atherosclerosis	-0.22	4.59E-09	0.41
					Myocardial infarction	-0.32	1.48E-11	0.94
					Coronary revascular.	-0.36	1.10E-12	0.93
					Statin medication	-0.61	5.26E-119	1
HNF4A	20:44413724:C:T	rs1800961	0.05	MODY, type I, AD	Cholelithiasis	0.38	1.27E-30	1
					Intrahepatic Cholestasis of Pregnancy	0.82	2.43E-09	1
AIRE	21:44294411:C:T	rs74203920	0.04	Autoimm. polyendocrinopathy syndrome AR, AD	Diabetic maculopathy	0.64	2.39E-09	0.99
					Type 1 diabetes	0.38	2.19E-10	0.96
					Diabetic retinopathy	0.48	9.48E-11	1
IFIH1	2:162267541:C:T	rs1990760	0.58	Aicardi-Goutieres AD;	Hypothyroidism	0.08	8.88E-11	0.62
				Singleton-Merten, AD	Disorders of the thyroid gland	0.07	5.43E-09	0.51
APOB	2:21041028:G:A	rs1367117	0.28	Hypercholesterolemia AD<br>Hypobetalipoproteinemia, AR	Disorders of lipoprotein metabolism	0.11	6.64E-12	0.51
ZAP70	2:97725153:C:T	rs145955907	0.019	Autoimmune disease, infantile, AR	Hypothyroidism	0.24	1.83E-08	0.43
LRBA	4:150277928:G:A	rs2290846	0.22	Immunodeficiency, with autoimmunity, AR	Cholelithiasis	0.12	1.31E-13	0.52
					Disorders of biliary tract and pancreas	0.1	2.19E-12	0.7
DBH	9:133636606:C:T	rs77273740	0.05	Orthostatic hypotension due to DBH deficiency, AR	Hypertensive diseases	-0.16	6.16E-13	0.99

Supplementary Table S10. Summary statistics of all coding variants in FinnGen release 4 (any additive association with p-value $<1x10^{-4}$).

Supplementary Notes.

1) Variants causing disease with recessive inheritance are at higher MAF in Finnish than Non-Finnish Europeans. In light of a global enrichment of deleterious variants in Finland[6,7] and the well-described “Finnish Disease Heritage”[8,9], we hypothesized that a set of known disease-causing variants should exist at higher frequencies in Finland. We thus compared minor allele frequencies (MAF) of 2419 unique variants that were listed in the disease variant database ClinVar[10] (version 2018) as pathogenic or likely pathogenic (likely pathogenic) in genes with only recessive inheritance annotated in the Online Mendelian Inheritance in Man, OMIM® (omim.org) between 10,824 Finnish (FIN) and 10,824 Non-Finnish Europeans (NFE) taken from the gnomAD population database[11] (Supplementary Figure S1). Concordant with a known lower rare variant diversity[9], only 554 likely pathogenic variants were present in FIN while 2169 were present in NFE at same sample sizes. As expected, however, observed likely pathogenic variants were at higher MAF in FIN (median MAF 9.2×10^{-5}) than NFE (median MAF = 4.7×10^{-5}, p-value = 6×10^{-7}, Wilcoxon rank test), with the difference particularly pronounced in 133 variants in Finnish disease heritage genes[8] (see Supplementary Figure S1). In FIN, 12% (67/554) of likely pathogenic variants were above MAF 0.001 (39% [217/554] above MAF 10^{-4}) compared to 2.5% (54/2169) in NFE (26% [559/2169] above MAF 10^{-4}).

2) Simulations of recessive and additive associations. We performed simulations of genotype and phenotype data to assess the output of our recessive and additive model in the presence of a strictly recessive or additive association. We simulated genotype and phenotype data aiming to resemble our own data. Specifically, we generated genotype counts of wildtype, heterozygotes and mutant homozygotes in 200,000 individuals of a variant at four different MAF thresholds following Hardy Weinberg Equilibrium (using R library HardyWeinberg[12]). We then generated random phenotype data of cases versus controls of a disease with a prevalence of 0.05 using the rbinom function in base R. We tested a recessive and an additive scenario. For the recessive scenario, we set the probability of homozygotes to develop the disease to 5x compared to wildtype and set heterozygote effect sizes to 1 corresponding to no heterozygous effect. For the additive scenario, we set the heterozygote effect to 1.5 and homozygous effect to 2.25 corresponding to a strictly additive effect on a logarithmic scale. We then performed logistic regression using the glm function in R with an additive and a recessive model, respectively.

For visualization and a table of results please see Extended Data Figure 2 and Supplementary Table S4. In summary, we find that if a simulated recessive effect reaches genomewide significance in the recessive model (recessive p-value <5x10e-8), the recessive p-value is 2 orders of magnitude below the additive p-value in 298,414/298,418 instances across 4x100,000 simulations. Thus, if truly recessive associations reach genomewide significance, we will likely identify them as recessive with our approach. Conversely, we find that for a simulated strictly additive effect in logarithmic space (heterozygous effect 1.5, homozygous effect 2.25; 100,000 simulations each at minor allele frequency thresholds 0.01, 0.02, 0.05, 0.1) recessive p-value is 2 orders of magnitude below the additive p-value in 0/400,000 simulations. We can thus with high confidence reject strict additivity when recessive p-value is 2 orders of magnitude below the additive p-value.

In an earlier version of this manuscript, we found in additional simulations that for increasing sizes of heterozygous effects the additive model's significance increased accordingly [13]. We found similar results for varying effect sizes and MAF.

3) Novel recessive disease associations. Here, we highlight two variants with large homozygous effects in genes with no previous disease associations, in *CASP7* associated with cataract, *C10orf90* associated with hearing loss and *EBAG9* associated with female infertility.

We would first like to present a missense variant in *CASP7* with a MAF of 0.015 in Finland (4x enriched in Finns) that we found associated with adult-onset cataract. The variant had large homozygous effects on the distinct phenotypes "other cataract" (p-value = $2x10^{-16}$, beta 4.8, most frequent ICD10 codes in 43 homozygotes: 13x secondary cataract and 7x presenile cataract) and "senile cataract" (p-value = $2x10^{-12}$, beta 2.1). While there were too few homozygotes in the UKBB we could replicate a genomewide significant effect on phenotype "all cataracts" in the additive model in UKBB (p-value = $5x10^{-12}$, beta 0.7) suggesting a potential heterozygous effect or homozygous effect of other variants in LD. There was also some evidence for an effect on "other cataracts" in heterozygous carriers in FinnGen (SAiGE GWAS p-value 0.003, beta 0.25). Age at first diagnosis was nominally significantly earlier in homozygotes (see Supplementary Figure 5). In the age group below 63 (mean retirement age in Finland) 50% of homozygotes (13/26) but only 3.4% (94/2750) of heterozygous and 3.1% (2710/87799) of wildtype carriers had already reported cataracts in our data. *CASP7* encodes Caspase-7, an executing enzyme in apoptosis (programmed cell death) [14]. In lens development, cell organelles are degraded to create transparent cells by a process very similar to

apoptosis. Further, deactivating apoptosis genes in mice (e.g. *Casp3*[15] or *Apaf-1* and *p53*[16]) has been shown to lead to cataract and there has been evidence for apoptosis involved in human non-congenital cataract[17,18]. *CASP7* is therefore a biologically plausible and to the best of our knowledge first Mendelian gene for adult-onset cataract, the leading cause of vision loss worldwide with no other effective treatment than surgery and therefore particularly affecting low/middle income countries (Source: WHO, 2012).

The missense variant in *C10orf90* was associated with sensorineural hearing loss also with Mendelian effects (recessive p-value = $5x10^{-12}$, beta 6.0; additive p-value > 10^{-4}), which we could replicate in the UKBB (recessive p-value $1x10^{-4}$, beta 56). The variant was 2.8x enriched in Finns with a MAF of 0.0083 in Finland. Homozygous carriers had significantly earlier onset of diseases: among 14k individuals diagnosed with hearing loss carriers of homozygous variants in *C10orf90* were first diagnosed at a mean age of 40±17 (mean±sd) compared to wildtype carriers (63±16, p-value $8*10^{-5}$, Wilcoxon-rank test). There is less known about the gene *C10orf90* which is also called fragile-site associated tumor suppressor (*FATS*)[19]. However, mutations in its most closely related paralog gene *ALMS1* cause the ciliopathy "Alstrom syndrome" with sensorineural hearing loss as one of its main symptoms[20]. As there is also some evidence for a role of *C10orf90* in cilia formation[21], it is plausible that *C10orf90* would also cause hearing via an effect on cilia. However, since there are no other symptoms in variant carriers beyond expectation apart from hearing loss, this is likely restricted to hearing loss and therefore distinct from *ALMS1*.

Next, we find a noncoding variant in *EBAG9* with a homozygous effect on female infertility. This is the only noncoding variant we investigated in our study but we believe it is the more likely causal candidate than the linked PTV variant in *PKHD1L1* we initially identified as it has a larger effect (beta 2.0 compared to 0.21), it is more likely causal after finemapping (PIP 0.2 while the PTV in *PKHD1L1* is not in the credible set) and *EBAG9* is a known estrogen responsive gene. 48 of 4,505 women (1.1%) with infertility in FinnGen are homozygous for the *EBAG9* variant. In FinnGen R6 data, we investigated the effect of the *EBAG9* variant on fertility in 106,732 women born between 1925 and 1975 for whom we had thus approximately the whole reproductive life span available. We find that 357 homozygotes of the *EBAG9* variant had fewer children (1.7±1.3 [mean±sd], p-value 0.0002, Wilcoxon rank test) than wildtypes (2.0±1.3) and a later age at first child (27.2±5.2 versus 28.3±5.5, p-value 0.01, Wilcoxon rank test). This effect is mainly driven by 71 *EBAG9* homozygotes

diagnosed with infertility as it is in the expected direction but not significant in 302 *EBAG9* homozygotes without the infertility diagnosis (mean 1.85 children, p-value 0.08, Wilcoxon rank test). Among all 7,980 women diagnosed with infertility 71 *EBAG9* homozygotes were more severely affected as they had fewer children (1.0±0.9 [mean±sd]) than other women with infertility diagnoses (1.5±1.2 [mean±sd]) p-value 0.005, Wilcoxon rank test) and had their first child significantly later (32.3±4.6 versus 30.0±5.6, p-value 0.005, Wilcoxon rank test).

4) Compound heterozygous effects. The genes *GJB2* and *EYS* harbored more than one pathogenic variant with significant recessive associations in our data so we sought to explore compound heterozygous (=comp-het) effects. We computed linkage disequilibrium (LD) between those variants with a Finnish population reference panel consisting of 2,244 high-coverage (30x) whole genome sequenced individuals[22]. LD between the two *GJB2* variants (r^2=0.002) was negligible but the two *EYS* variants were in strong LD (r^2=0.47) so we could not investigate comp-het effects for *EYS*. In *GJB2,* we found early onset sensorineural hearing loss in 100 *GJB2* comp-hets, i.e. individuals with one pathogenic missense and one pLoF *GJB2* variant (p-value $6x10^{-28}$, HR 6.1, coxph) that each also had effects in homozygous state. In agreement with the variants' different homozygous effect sizes, the effect of comp-het was in between the homozygous pLoF (HR 15, p-value $7x10^{-37}$) and homozygous missense variants' effect sizes (HR 3.9, p-value $8x10^{-15}$) (see Figure 3C). We also tested the effects of another likely pathogenic missense variant in *GJB2.* LD with the other *GJB2* variants was negligible (r^2~0.00 with both). That variant was at a much lower MAF of 0.004. Therefore, our dataset included only 3 individuals in homozygous state, 4 in compound heterozygous state with the pLoF *GJB2* variant and 5 in compound heterozygous state with the other missense *GJB2* variant. Combined, all 12 variants were associated with early onset sensorineural hearing loss with similar effect as the other pathogenic variants in *GJB2* (p-value $3x10^{-12}$, HR 11.8, coxph). We also investigated comp-het effects in *CERKL* as there was an additional known pathogenic pLoF variant in our data chr2:181656814:C:A (hg38) at low frequency (MAF 0.0019) which was thus not well-powered to reach significance in homozygous state in the pheWAS. When excluding individuals with homozygous and comp-het *CERKL* variants we did not find a heterozygous effect of any of the pathogenic variants on the phenotype of retinal dystrophy anymore suggesting comp-het effects drove the initial nominally significant heterozygous effect.

5) Known disease causing variants in FinnGen. We studied 15,140 variants that were annotated in ClinVar[10] in 176,899 Finns in the FinnGen research project. These included 311 coding variants that were annotated as likely pathogenic, 147 of them by multiple submitters or an expert panel (corresponds to ClinVar review status of 2-4 out of 4 possible stars) which we thus describe as being annotated as likely pathogenic with "high confidence". 298 further variants had "conflicting interpretations of pathogenicity" but were classified as likely pathogenic by at least one submitter. 3.4% of study participants carried at least one of ten high confidence likely pathogenic variant in one of nine genes causing disease with dominant inheritance according to OMIM. For 6 out of the 10 likely pathogenic variants we found expected genomewide significant disease associations (see also Supplementary Table S1) in the genes *FLG* (atopic dermatitis, 2690 and 1050 heterozygotes, respectively), *MYOC* (glaucoma, 1023 heterozygotes), *PALB2* (breast cancer, 512 heterozygotes), *JAK2* (Polycythaemia vera, 194 heterozygotes) and *MYBPC3* (Hypertrophic cardiomyopathy, 116 heterozygotes). Accordingly, we did not find genomewide significant associations for 4 of 10 high confidence likely pathogenic variants of which *CHEK2* was genomewide significant for breast cancer in the later FinnGen datafreeze R6. Variants in *COL5A2, G6PD* and *PKD1* were not significant in later releases. For *G6PD* this may be expected as the variant is rare (58 hemizygous males) and functionally predicted to lead to Glucose-6-phosphate dehydrogenase activity of 15 to 25 percent of wildtype (Cappellini 1994, Cappellini 1995, De Vita 1989, Frigerio 1994, Lenzerini 1969, Rattazzi 1969), thus characteristic of a moderate (Class III) deficiency where hemolysis is only triggered by stressors. This rare and moderate effect may thus be underpowered to be detected by our EHR data. For variants in *COL5A2* and *PKD1* we would expect at least nominally significant disease associations in later FinnGen releases, given they contain > 60,000 individuals with joint disorders and >1,000 individuals with cystic kidney disease. The fact that there are only two submitters for the variant in *COL5A2* and one submitter classifying the variant in *PKD1* as VUS in a later ClinVar release adds to the suspicion that they may in fact not be truly disease associated.

All of 0.41% of participants (n=726) carried one of 98 high confidence likely pathogenic variants in homozygous state in one of 86 disease genes with known recessive inheritance, 41.8% were heterozygous carriers. Most frequent homozygous mutants were in the genes *BTD* (partial biotinidase deficiency, 523 homozygotes), *GJB2* (nonsyndromic sensorineural hearing loss, 113 homozygotes) and *SERPINA1* (Alpha-1-antitrypsin deficiency, 77 homozygotes). We found

expected homozygous phenotype associations of *GJB2* and *SERPINA1* variants with genomewide significance. We were however surprised by the lack of genomewide signifiant disease associations for the known pathogenic variant [*BTD*:p.(Asp444His)] linked to biotinidase deficiency. *BTD*:p.(Asp444His) has been reported as pathogenic for biotinidase deficiency by 17 submitters in ClinVar and experimentally led to a ~25% reduction in biotinidase activity[23]. Homozygotes for *BTD*:p.(Asp444His) are thus expected to have ca. 50% of normal serum biotinidase activitiy. As partial biotinidase deficiency is however characterized by 10%-30% of normal serum biotinidase activity[24] the lack of disease associations above chance expectation for *BTD*:p.(Asp444His) homozygotes makes sense. We can thus empirically confirm the experimental hypothesis that the variant should only cause disease when compound heterozygous with a variant leading to near-zero biotinidase activity.

We also demonstrate how our results help to verify disease associations of variants with low sample sizes. One example is a missense variant in *SPINK1* with conflicting interpretations of likely benign (1x), likely pathogenic (8x) and VUS (3x) on ClinVar. In our data, it is the only coding variant, most significant and most likely causal variant (finemapping probability 0.19 in FinnGen datafreeze 8) at the respective locus associated with alcohol-induced acute and chronic pancreatitis as well as acute and chronic pancreatitis more generally with odd's ratios between 2.8-8.5. Its inheritance is best described as semidominant with an additive genotype dosage effect. Another example is a variant in *SOD1* with conflicting interpretations of likely benign (1x), likely pathogenic (5x) and VUS (4x) on ClinVar. In our data, we find a large homozygous effect on motor neuron disease with odd's ratio > 100 while not detecting a heterozygous effect which we can confirm with R6 data in a total of 8 cases. We thus classify inheritance as recessive. We are currently preparing the full list of associations of unclear disease variants for submission to ClinVar. We can thus show that we are well-powered to identify known associations across a wide range of disease phenotypes in our dataset. In addition, it can serve as a resource to verify or falsify previously described disease associations. For verification purposes the statistical finemapping can be additionally helpful to identify associations' causal variants.

6) Benign and likely benign (likely benign) variants in FinnGen. We studied effects of variants listed in ClinVar [10] as likely benign. Those are regarded as "not the cause for a patient's disorder" ACMG [25]. However, we found more phenotype associations than expected in likely benign ClinVar variants as a group.

To test whether these might potentially just correlate with actual causal variants in linkage disequilibrium we assessed how often they were included in the 95%-credible set (cs) for a given GWAS locus [5]. We found on the contrary, that 142 likely benign missense variants were slightly more often in the 95%-cs than 307 missense variants not annotated in ClinVar (Fisher's Exact test, p-value 0.05, OR 1.5, 95%-CI 1.0-2.4). 16 represented even the most likely causal variant for a GWAS locus. The ClinVar annotation labeling these variants as likely benign was above average quality (1.6; 7 of the 16 likely benign top causal variants had a one star and 9 of 16 a two-star review status in ClinVar) compared to an average 1.2 stars for all likely benign variants in ClinVar (range: zero to three stars). Thus, poor quality of ClinVar classification as likely benign cannot explain why so many benign variants in our dataset are disease associated.

Expectedly, the disease phenotypes in FinnGen – though often related - are mostly different from the known OMIM phenotypes of the respective genes. Nevertheless, disease associations of likely benign variants may still be counterintuitive as they are often interpreted as generally disease-irrelevant [26,27]. How could we then explain global disease associations for them? Firstly, it is plausible that the frequent diagnostic sequencing and analysis of disease-associated genes with high clinical interest also results in submission of many likely benign variants to ClinVar for those genes. Hypothesizing that genes with many disease-causing variants would therefore also get many ClinVar submissions of likely benign variants, we found indeed a positive correlation of the number of likely pathogenic and likely benign variants per gene in ClinVar (r^2 0.41, rho 0.07, respective p $<10^{-16}$). Genes with many disease-causing variants - particularly relevant to human disease - may be more likely involved in additional diseases than disease-irrelevant genes [28]. In line with that we found indeed that likely benign ClinVar missense variants had more significant phenotype associations than missense variants in different genes but that signal disappeared mostly when missense variants were matched to the same genes. In addition, likely benign variants are of significantly higher MAF (0.07±0.12, mean, sd) than other ClinVar variants (0.01±0.05) in FinnGen which is expected as MAF is a criterion for labeling a variant as likely benign according to official ACMG guidelines [25]. So we are well-powered to find significant disease associations for likely benign variants, particularly in a bottleneck population like Finland that enriches disease variants to higher frequencies [9]. Of note, when investigating unique ClinVar variants in the 95%-cs, 19 out of 67 likely benign ClinVar variants had protective effects on disease, while only 1 out of 27 ClinVar risk variants (i.e. likely pathogenic, conflicting or risk factor variants) was protective (Fisher's

Exact test, p-value 0.01 OR 10, 95%-CI 1.4-443). While their identified effects are more often protective than for known risk variants the majority of associations increase disease risk also in benign variants! In conclusion, we think it is important to keep in mind that variants labeled as likely benign are not generally neutral and can still contribute to disease.

References

1 Grubb, R. Correlation between Lewis blood group and secretor character in man. *Nature* **162**, 933, doi:10.1038/162933a0 (1948).

2 Laine, A. P. *et al.* Two insulin gene single nucleotide polymorphisms associated with type 1 diabetes risk in the Finnish and Swedish populations. *Dis Markers* **23**, 139-145, doi:10.1155/2007/574363 (2007).

3 Howson, J. M., Walker, N. M., Smyth, D. J., Todd, J. A. & Type, I. D. G. C. Analysis of 19 genes for association with type I diabetes in the Type I Diabetes Genetics Consortium families. *Genes Immun* **10 Suppl 1**, S74-84, doi:10.1038/gene.2009.96 (2009).

4 Bycroft, C. *et al.* The UK Biobank resource with deep phenotyping and genomic data. *Nature* **562**, 203-209, doi:10.1038/s41586-018-0579-z (2018).

5 Wang, G., Sarkar, A., Carbonetto, P. & Stephens, M. A simple new approach to variable selection in regression, with application to genetic fine mapping. *Journal of the Royal Statistical Society: Series B (Statistical Methodology)* **82**, 1273-1300, doi:https://doi.org/10.1111/rssb.12388 (2020).

6 Locke, A. E. *et al.* Exome sequencing of Finnish isolates enhances rare-variant association power. *Nature* **572**, 323-328, doi:10.1038/s41586-019-1457-z (2019).

7 Lim, E. T. *et al.* Distribution and medical impact of loss-of-function variants in the Finnish founder population. *PLoS Genet* **10**, e1004494, doi:10.1371/journal.pgen.1004494 (2014).

8 Polvi, A. *et al.* The Finnish disease heritage database (FinDis) update-a database for the genes mutated in the Finnish disease heritage brought to the next-generation sequencing era. *Hum Mutat* **34**, 1458-1466, doi:10.1002/humu.22389 (2013).

9 Peltonen, L., Jalanko, A. & Varilo, T. Molecular genetics of the Finnish disease heritage. *Hum Mol Genet* **8**, 1913-1923, doi:10.1093/hmg/8.10.1913 (1999).

10 Landrum, M. J. *et al.* ClinVar: public archive of interpretations of clinically relevant variants. *Nucleic Acids Res* **44**, D862-868, doi:10.1093/nar/gkv1222 (2016).

11 Karczewski, K. J. *et al.* The mutational constraint spectrum quantified from variation in 141,456 humans. *Nature* **581**, 434-443, doi:10.1038/s41586-020-2308-7 (2020).

12 Graffelman, J. Exploring Diallelic Genetic Markers: The HardyWeinberg Package. *2015* **64**, 23, doi:10.18637/jss.v064.i03 (2015).

13 Heyne, H. *et al.* Mono-and bi-allelic effects of coding variants on disease in 176,899 Finns. *medRxiv* (2021).

14 Lakhani, S. A. *et al.* Caspases 3 and 7: key mediators of mitochondrial events of apoptosis. *Science* **311**, 847-851, doi:10.1126/science.1115035 (2006).

15 Zandy, A. J., Lakhani, S., Zheng, T., Flavell, R. A. & Bassnett, S. Role of the executioner caspases during lens development. *J Biol Chem* **280**, 30263-30272, doi:10.1074/jbc.M504007200 (2005).

16 Guo, Z., Yikang, S., Yoshida, H., Mak, T. W. & Zacksenhaus, E. Inactivation of the retinoblastoma tumor suppressor induces apoptosis protease-activating factor-1 dependent and independent apoptotic pathways during embryogenesis. *Cancer Res* **61**, 8395-8400 (2001).
17 Li, W. C. *et al.* Lens epithelial cell apoptosis appears to be a common cellular basis for non-congenital cataract development in humans and animals. *J Cell Biol* **130**, 169-181, doi:10.1083/jcb.130.1.169 (1995).
18 Yan, Q., Liu, J. P. & Li, D. W. Apoptosis in lens development and pathology. *Differentiation* **74**, 195-211, doi:10.1111/j.1432-0436.2006.00068.x (2006).
19 Yan, S. *et al.* FATS is an E2-independent ubiquitin ligase that stabilizes p53 and promotes its activation in response to DNA damage. *Oncogene* **33**, 5424-5433, doi:10.1038/onc.2013.494 (2014).
20 Alstrom, C. H., Hallgren, B., Nilsson, L. B. & Asander, H. Retinal degeneration combined with obesity, diabetes mellitus and neurogenous deafness: a specific syndrome (not hitherto described) distinct from the Laurence-Moon-Bardet-Biedl syndrome: a clinical, endocrinological and genetic examination based on a large pedigree. *Acta Psychiatr Neurol Scand Suppl* **129**, 1-35 (1959).
21 Knorz, V. J. *et al.* Centriolar association of ALMS1 and likely centrosomal functions of the ALMS motif-containing proteins C10orf90 and KIAA1731. *Mol Biol Cell* **21**, 3617-3629, doi:10.1091/mbc.E10-03-0246 (2010).
22 Mitt, M. *et al.* Improved imputation accuracy of rare and low-frequency variants using population-specific high-coverage WGS-based imputation reference panel. *Eur J Hum Genet* **25**, 869-876, doi:10.1038/ejhg.2017.51 (2017).
23 Norrgard, K. J. *et al.* Mutation (Q456H) is the most common cause of profound biotinidase deficiency in children ascertained by newborn screening in the United States. *Biochem Mol Med* **61**, 22-27, doi:10.1006/bmme.1997.2597 (1997).
24 Wolf, B. Biotinidase Deficiency. *GeneReviews®* **1993-2021** (2000 Mar 24 [Updated 2016 Jun 9]). <https://www.ncbi.nlm.nih.gov/books/NBK1322/>.
25 Richards, S. *et al.* Standards and guidelines for the interpretation of sequence variants: a joint consensus recommendation of the American College of Medical Genetics and Genomics and the Association for Molecular Pathology. *Genet Med* **17**, 405-424, doi:10.1038/gim.2015.30 (2015).
26 Lek, M. *et al.* Analysis of protein-coding genetic variation in 60,706 humans. *Nature* **536**, 285-291, doi:10.1038/nature19057 (2016).
27 Yang, S. *et al.* Sources of discordance among germ-line variant classifications in ClinVar. *Genet Med* **19**, 1118-1126, doi:10.1038/gim.2017.60 (2017).
28 Freund, M. K. *et al.* Phenotype-Specific Enrichment of Mendelian Disorder Genes near GWAS Regions across 62 Complex Traits. *Am J Hum Genet* **103**, 535-552, doi:10.1016/j.ajhg.2018.08.017 (2018).

FinnGen members

Full Name	Affiliation	E-mail	Role 1	Role 2
Aarno Palotie	Institute for Molecular Medicine Finland (FIMM), HiLIFE, University of Helsinki, Helsinki, Finland; Broad Institute of MIT and Harvard; Massachusetts General Hospital	aarno.palotie@helsinki.fi	Steering Committee	Steering Committee
Mark Daly	Institute for Molecular Medicine Finland (FIMM), HiLIFE, University of Helsinki, Helsinki, Finland; Broad Institute of MIT and Harvard; Massachusetts General Hospital	mark.daly@helsinki.fi	Steering Committee	Steering Committee
Bridget Riley-Gills	Abbvie, Chicago, IL, United States	bridget.rileygillis@abbvie.com	Steering Committee	**Pharmaceutical companies**
Howard Jacob	Abbvie, Chicago, IL, United States	howard.jacob@abbvie.com	Steering Committee	**Pharmaceutical companies**
Dirk Paul	Astra Zeneca, Cambridge, United Kingdom	dirk.paul@astrazeneca.com	Steering Committee	**Pharmaceutical companies**
Athena Matakidou	Astra Zeneca, Cambridge, United Kingdom	athena.x.matakidou@gsk.com	Steering Committee	**Pharmaceutical companies**
Adam Platt	Astra Zeneca, Cambridge, United Kingdom	adam.platt@astrazeneca.com	Steering Committee	**Pharmaceutical companies**
Heiko Runz	Biogen, Cambridge, MA, United States	heiko.runz@biogen.com	Steering Committee	**Pharmaceutical companies**
Sally John	Biogen, Cambridge, MA, United States	sally.john@biogen.com	Steering Committee	**Pharmaceutical companies**
George Okafo	Boehringer Ingelheim, Ingelheim am Rhein, Germany	george.okafo@boehringer-ingelheim.com	Steering Committee	**Pharmaceutical companies**
Nathan Lawless	Boehringer Ingelheim, Ingelheim am Rhein, Germany	nathan.lawless@boehringer-ingelheim.com	Steering Committee	**Pharmaceutical companies**
Heli Salminen-Mankonen	Boehringer Ingelheim, Ingelheim am Rhein, Germany	heli.salminen-mankonen@boehringer-ingelheim.com	Steering Committee	**Pharmaceutical companies**
Robert Plenge	Bristol Myers Squibb, New York, NY, United States	robert.plenge@bms.com	Steering Committee	**Pharmaceutical companies**
Joseph Maranville	Bristol Myers Squibb, New York, NY, United States	joseph.maranville@bms.com	Steering Committee	**Pharmaceutical companies**
Mark McCarthy	Genentech, San Francisco, CA, United States	mccarthy.mark@gene.com	Steering Committee	**Pharmaceutical companies**
Julie Hunkapiller	Genentech, San Francisco, CA, United States	hunkapiller.julie@gene.com	Steering Committee	**Pharmaceutical companies**
Margaret G. Ehm	GlaxoSmithKline, Collegeville, PA, United States	meg.g.ehm@gsk.com	Steering Committee	**Pharmaceutical companies**
Kirsi Auro	GlaxoSmithKline, Espoo, Finland	kirsi.m.auro@gsk.com	Steering Committee	**Pharmaceutical companies**
Simonne Longerich	Merck, Kenilworth, NJ, United States	simonne.longerich@merck.com	Steering Committee	**Pharmaceutical companies**
Caroline Fox	Merck, Kenilworth, NJ, United States	caroline.fox@merck.com	Steering Committee	**Pharmaceutical companies**
Anders Mälarstig	Pfizer, New York, NY, United States	anders.malarstig@pfizer.com	Steering Committee	**Pharmaceutical companies**
Katherine Klinger	Translational Sciences, Sanofi R&D, Framingham, MA, USA	katherine.klinger@sanofi.com	Steering Committee	**Pharmaceutical companies**
Deepak Raipal	Translational Sciences, Sanofi R&D, Framingham, MA, USA	deepak.rajpal@sanofi.com	Steering Committee	**Pharmaceutical companies**
Eric Green	Maze Therapeutics, San Francisco, CA, United States	egreen@mazetx.com	Steering Committee	**Pharmaceutical companies**
Robert Graham	Maze Therapeutics, San Francisco, CA, United States	rgraham@mazetx.com	Steering Committee	**Pharmaceutical companies**
Robert Yang	Janssen Biotech, Beerse, Belgium	ryang31@its.jnj.com	Steering Committee	**Pharmaceutical companies**
Chris O´Donnell	Novartis Institutes for BioMedical Research, Cambridge, MA, United States	chris.odonnell@novartis.com	Steering Committee	**Pharmaceutical companies**
Tomi P. Mäkelä	HiLIFE, University of Helsinki, Finland, Finland	tomi.makela@helsinki.fi	Steering Committee	**University of Helsinki & Biobanks**
Jaakko Kaprio	Institute for Molecular Medicine Finland (FIMM), HiLIFE, University of Helsinki, Helsinki, Finland	jaakko.kaprio@helsinki.fi	Steering Committee	**University of Helsinki & Biobanks**
Petri Virolainen	Auria Biobank / University of Turku / Hospital District of Southwest Finland, Turku, Finland	petri.virolainen@tyks.fi	Steering Committee	**University of Helsinki & Biobanks**
Antti Hakanen	Auria Biobank / University of Turku / Hospital District of Southwest Finland, Turku, Finland	antti.hakanen@tyks.fi	Steering Committee	**University of Helsinki & Biobanks**
Terhi Kilpi	THL Biobank / Finnish Institute for Health and Welfare (THL), Helsinki, Finland	terhi.kilpi@thl.fi	Steering Committee	**University of Helsinki & Biobanks**
Markus Perola	THL Biobank / Finnish Institute for Health and Welfare (THL), Helsinki, Finland	markus.perola@thl.fi	Steering Committee	**University of Helsinki & Biobanks**
Jukka Partanen	Finnish Red Cross Blood Service / Finnish Hematology Registry and Clinical Biobank, Helsinki, Finland	jukka.partanen@veripalvelu.fi	Steering Committee	**University of Helsinki & Biobanks**
Anne Pitkäranta	Helsinki Biobank / Helsinki University and Hospital District of Helsinki and Uusimaa, Helsinki	anne.pitkaranta@hus.fi	Steering Committee	**University of Helsinki & Biobanks**
Taneli Raivio	Helsinki Biobank / Helsinki University and Hospital District of Helsinki and Uusimaa, Helsinki	taneli.raivio@hus.fi	Steering Committee	**University of Helsinki & Biobanks**
Raisa Serpi	Northern Finland Biobank Borealis / University of Oulu / Northern Ostrobothnia Hospital District, Oulu, Finland	raisa.serpi@ppshp.fi	Steering Committee	**University of Helsinki & Biobanks**
Tarja Laitinen	Finnish Clinical Biobank Tampere / University of Tampere / Pirkanmaa Hospital District, Tampere, Finland	tarja.laitinen@pshp.fi	Steering Committee	**University of Helsinki & Biobanks**
Veli-Matti Kosma	Biobank of Eastern Finland / University of Eastern Finland / Northern Savo Hospital District, Kuopio, Finland	veli-matti.kosma@uef.fi	Steering Committee	**University of Helsinki & Biobanks**
Jari Laukkanen	Central Finland Biobank / University of Jyväskylä / Central Finland Health Care District, Jyväskylä, Finland	jari.laukkanen@ksshp.fi	Steering Committee	**University of Helsinki & Biobanks**
Marco Hautalahti	FINBB - Finnish biobank cooperative	marco.hautalahti@finbb.fi	Steering Committee	**University of Helsinki & Biobanks**
Outi Tuovila	Business Finland, Helsinki, Finland	outi.tuovila@businessfinland.fi	Steering Committee	**Other Experts/ Non-Voting Members**
Raimo Pakkanen	Business Finland, Helsinki, Finland	raimo.pakkanen@businessfinland.fi	Steering Committee	**Other Experts/ Non-Voting Members**
Jeffrey Waring	Abbvie, Chicago, IL, United States	jeff.waring@abbvie.com	Scientific Committee	**Pharmaceutical companies**
Bridget Riley-Gillis	Abbvie, Chicago, IL, United States	bridget.rileygillis@abbvie.com	Scientific Committee	**Pharmaceutical companies**
Fedik Rahimov	Abbvie, Chicago, IL, United States	fedik.rahimov@abbvie.com	Scientific Committee	**Pharmaceutical companies**
Ioanna Tachmazidou	Astra Zeneca, Cambridge, United Kingdom	ioanna.tachmazidou@astrazeneca.com	Scientific Committee	**Pharmaceutical companies**
Chia-Yen Chen	Biogen, Cambridge, MA, United States	chiayen.chen@biogen.com	Scientific Committee	**Pharmaceutical companies**
Heiko Runz	Biogen, Cambridge, MA, United States	heiko.runz@biogen.com	Scientific Committee	**Pharmaceutical companies**
Zhihao Ding	Boehringer Ingelheim, Ingelheim am Rhein, Germany	zhihao.ding@boehringer-ingelheim.com	Scientific Committee	**Pharmaceutical companies**
Marc Jung	Boehringer Ingelheim, Ingelheim am Rhein, Germany	marc_oliver.jung@boehringer-ingelheim.com	Scientific Committee	**Pharmaceutical companies**
Shameek Biswas	Bristol Myers Squibb, New York, NY, United States	Shameek.Biswas@bms.com	Scientific Committee	**Pharmaceutical companies**
Rion Pendergrass	Genentech, San Francisco, CA, United States	penders2@gene.com	Scientific Committee	**Pharmaceutical companies**
Julie Hunkapiller	Genentech, San Francisco, CA, United States	hunkapiller.julie@gene.com	Scientific Committee	**Pharmaceutical companies**
Margaret G. Ehm	GlaxoSmithKline, Collegeville, PA, United States	meg.g.ehm@gsk.com	Scientific Committee	**Pharmaceutical companies**
David Pulford	GlaxoSmithKline, Stevenage, United Kingdom	david.x.pulford@gsk.com	Scientific Committee	**Pharmaceutical companies**
Neha Raghavan	Merck, Kenilworth, NJ, United States	neha.raghavan@merck.com	Scientific Committee	**Pharmaceutical companies**
Adriana Huertas-Vazquez	Merck, Kenilworth, NJ, United States	adriana.huertas.vazquez@merck.com	Scientific Committee	**Pharmaceutical companies**
Jae-Hoon Sul	Merck, Kenilworth, NJ, United States	jae.hoon.sul@merck.com	Scientific Committee	**Pharmaceutical companies**
Anders Mälarstig	Pfizer, New York, NY, United States	anders.malarstig@pfizer.com	Scientific Committee	**Pharmaceutical companies**
Xinli Hu	Pfizer, New York, NY, United States	xinli.hu@pfizer.com	Scientific Committee	**Pharmaceutical companies**
Katherine Klinger	Translational Sciences, Sanofi R&D, Framingham, MA, USA	katherine.klinger@sanofi.com	Scientific Committee	**Pharmaceutical companies**
Robert Graham	Maze Therapeutics, San Francisco, CA, United States	rgraham@mazetx.com	Scientific Committee	**Pharmaceutical companies**
Eric Green	Maze Therapeutics, San Francisco, CA, United States	egreen@mazetx.com	Scientific Committee	**Pharmaceutical companies**
Sahar Mozaffari	Maze Therapeutics, San Francisco, CA, United States	smozaffari@mazetx.com	Scientific Committee	**Pharmaceutical companies**
Dawn Waterworth	Janssen Research & Development, LLC, Spring House, PA, United States	dwaterwo@its.jnj.com	Scientific Committee	**Pharmaceutical companies**
Nicole Renaud	Novartis Institutes for BioMedical Research, Cambridge, MA, United States	nicole.renaud@novartis.com	Scientific Committee	**Pharmaceutical companies**
Ma´en Obeidat	Novartis Institutes for BioMedical Research, Cambridge, MA, United States	maen.obeidat@novartis.com	Scientific Committee	**Pharmaceutical companies**
Samuli Ripatti	Institute for Molecular Medicine Finland (FIMM), HiLIFE, University of Helsinki, Helsinki, Finland	samuli.ripatti@helsinki.fi	Scientific Committee	**University of Helsinki & Biobanks**
Johanna Schleutker	Auria Biobank / Univ. of Turku / Hospital District of Southwest Finland, Turku, Finland	johanna.schleutker@utu.fi	Scientific Committee	**University of Helsinki & Biobanks**
Markus Perola	THL Biobank / Finnish Institute for Health and Welfare (THL), Helsinki, Finland	markus.perola@thl.fi	Scientific Committee	**University of Helsinki & Biobanks**
Mikko Arvas	Finnish Red Cross Blood Service / Finnish Hematology Registry and Clinical Biobank, Helsinki, Finland	mikko.arvas@veripalvelu.fi	Scientific Committee	**University of Helsinki & Biobanks**
Olli Carpén	Helsinki Biobank / Helsinki University and Hospital District of Helsinki and Uusimaa, Helsinki	olli.carpen@helsinki.fi	Scientific Committee	**University of Helsinki & Biobanks**
Reetta Hinttala	Northern Finland Biobank Borealis / University of Oulu / Northern Ostrobothnia Hospital District, Oulu, Finland	reetta.hinttala@oulu.fi	Scientific Committee	**University of Helsinki & Biobanks**
Johannes Kettunen	Northern Finland Biobank Borealis / University of Oulu / Northern Ostrobothnia Hospital District, Oulu, Finland	johannes.kettunen@oulu.fi	Scientific Committee	**University of Helsinki & Biobanks**
Arto Mannermaa	Biobank of Eastern Finland / University of Eastern Finland / Northern Savo Hospital District, Kuopio, Finland	arto.mannermaa@uef.fi	Scientific Committee	**University of Helsinki & Biobanks**
Katriina Aalto-Setälä	Faculty of Medicine and Health Technology, Tampere University, Tampere, Finland	katriina.aalto-setala@tuni.fi	Scientific Committee	**University of Helsinki & Biobanks**
Mika Kähönen	Finnish Clinical Biobank Tampere / University of Tampere / Pirkanmaa Hospital District, Tampere, Finland	mika.kahonen@uta.fi	Scientific Committee	**University of Helsinki & Biobanks**
Jari Laukkanen	Central Finland Biobank / University of Jyväskylä / Central Finland Health Care District, Jyväskylä, Finland	jari.laukkanen@ksshp.fi	Scientific Committee	**University of Helsinki & Biobanks**
Johanna Mäkelä	FINBB - Finnish biobank cooperative	johanna.makela@finbb.fi	Scientific Committee	**University of Helsinki & Biobanks**
Reetta Kälviäinen	Northern Savo Hospital District, Kuopio, Finland	reetta.kalviainen@kuh.fi	Clinical Groups	**Neurology Group**
Valtteri Julkunen	Northern Savo Hospital District, Kuopio, Finland	valtteri.julkunen@kuh.fi	Clinical Groups	**Neurology Group**
Hilkka Soininen	Northern Savo Hospital District, Kuopio, Finland	hilkka.soininen@uef.fi	Clinical Groups	**Neurology Group**
Anne Remes	Northern Ostrobothnia Hospital District, Oulu, Finland	anne.remes@oulu.fi	Clinical Groups	**Neurology Group**
Mikko Hiltunen	University of Eastern Finland, Kuopio, Finland	mikko.hiltunen@uef.fi	Clinical Groups	**Neurology Group**
Jukka Peltola	Pirkanmaa Hospital District, Tampere, Finland	jukka.peltola@pshp.fi	Clinical Groups	**Neurology Group**
Minna Raivio	Hospital District of Helsinki and Uusimaa, Helsinki, Finland	minna.raivio@geri.fi	Clinical Groups	**Neurology Group**
Pentti Tienari	Hospital District of Helsinki and Uusimaa, Helsinki, Finland	pentti.tienari@hus.fi	Clinical Groups	**Neurology Group**
Juha Rinne	Hospital District of Southwest Finland, Turku, Finland	juha.rinne@tyks.fi	Clinical Groups	**Neurology Group**
Roosa Kallionpää	Hospital District of Southwest Finland, Turku, Finland	roosa.kallionpaa@tyks.fi	Clinical Groups	**Neurology Group**
Juulia Partanen	Institute for Molecular Medicine Finland, HiLIFE, University of Helsinki, Finland	juulia.partanen@helsinki.fi	Clinical Groups	**Neurology Group**
Ali Abbasi	Abbvie, Chicago, IL, United States	ali.abbasi@abbvie.com	Clinical Groups	**Neurology Group**
Adam Ziemann	Abbvie, Chicago, IL, United States	adam.ziemann@abbvie.com	Clinical Groups	**Neurology Group**
Nizar Smaoui	Abbvie, Chicago, IL, United States	nizar.smaoui@abbvie.com	Clinical Groups	**Neurology Group**

Anne Lehtonen	Abbvie, Chicago, IL, United States	anne.lehtonen@abbvie.com	Clinical Groups	**Neurology Group**
Susan Eaton	Biogen, Cambridge, MA, United States	susan.eaton@biogen.com	Clinical Groups	**Neurology Group**
Heiko Runz	Biogen, Cambridge, MA, United States	heiko.runz@biogen.com	Clinical Groups	**Neurology Group**
Sanni Lahdenperä	Biogen, Cambridge, MA, United States	sanni.lahdenpera@biogen.com	Clinical Groups	**Neurology Group**
Shameek Biswas	Bristol Myers Squibb, New York, NY, United States	shameek.biswas@bms.com	Clinical Groups	**Neurology Group**
Julie Hunkapiller	Genentech, San Francisco, CA, United States	hunkapiller.julie@gene.com	Clinical Groups	**Neurology Group**
Natalie Bowers	Genentech, San Francisco, CA, United States	bowersn1@gene.com	Clinical Groups	**Neurology Group**
Edmond Teng	Genentech, San Francisco, CA, United States	teng.edmond@gene.com	Clinical Groups	**Neurology Group**
Rion Pendergrass	Genentech, San Francisco, CA, United States	penders2@gene.com	Clinical Groups	**Neurology Group**
Fanli Xu	GlaxoSmithKline, Brentford, United Kingdom	chun-fang.2.xu@gsk.com	Clinical Groups	**Neurology Group**
David Pulford	GlaxoSmithKline, Stevenage, United Kingdom	david.x.pulford@gsk.com	Clinical Groups	**Neurology Group**
Kirsi Auro	GlaxoSmithKline, Espoo, Finland	kirsi.m.auro@gsk.com	Clinical Groups	**Neurology Group**
Laura Addis	GlaxoSmithKline, Brentford, United Kingdom	laura.x.addis@gsk.com	Clinical Groups	**Neurology Group**
John Eicher	GlaxoSmithKline, Brentford, United Kingdom	john.d.eicher@gsk.com	Clinical Groups	**Neurology Group**
Qingqin S Li	Janssen Research & Development, LLC, Titusville, NJ 08560, United States	QLi2@its.jnj.com	Clinical Groups	**Neurology Group**
Karen He	Janssen Research & Development, LLC, Spring House, PA, United States	khe2@its.jnj.com	Clinical Groups	**Neurology Group**
Ekaterina Khramtsova	Janssen Research & Development, LLC, Spring House, PA, United States	ekhramts@its.jnj.com	Clinical Groups	**Neurology Group**
Neha Raghavan	Merck, Kenilworth, NJ, United States	neha.raghavan@merck.com	Clinical Groups	**Neurology Group**
Martti Färkkilä	Hospital District of Helsinki and Uusimaa, Helsinki, Finland	martti.farkkila@hus.fi	Clinical Groups	**Gastroenterology Group**
Jukka Koskela	Hospital District of Helsinki and Uusimaa, Helsinki, Finland	jukka.koskela@helsinki.fi	Clinical Groups	**Gastroenterology Group**
Sampsa Pikkarainen	Hospital District of Helsinki and Uusimaa, Helsinki, Finland	sampsa.pikkarainen@hus.fi	Clinical Groups	**Gastroenterology Group**
Airi Jussila	Pirkanmaa Hospital District, Tampere, Finland	airi.jussila@pshp.fi	Clinical Groups	**Gastroenterology Group**
Katri Kaukinen	Pirkanmaa Hospital District, Tampere, Finland	katri.kaukinen@tuni.fi	Clinical Groups	**Gastroenterology Group**
Timo Blomster	Northern Ostrobothnia Hospital District, Oulu, Finland	timo.blomster@ppshp.fi	Clinical Groups	**Gastroenterology Group**
Mikko Kiviniemi	Northern Savo Hospital District, Kuopio, Finland	mikko.kiviniemi@kuh.fi	Clinical Groups	**Gastroenterology Group**
Markku Voutilainen	Hospital District of Southwest Finland, Turku, Finland	markku.voutilainen@tyks.fi	Clinical Groups	**Gastroenterology Group**
Mark Daly	Institute for Molecular Medicine, Finland (FIMM), HiLIFE, University of Helsinki, Helsinki, Finland; Broad Institute of MIT and Harvard; Massachusetts General Hospital	mark.daly@helsinki.fi	Clinical Groups	**Gastroenterology Group**
Ali Abbasi	Abbvie, Chicago, IL, United States	ali.abbasi@abbvie.com	Clinical Groups	**Gastroenterology Group**
Jeffrey Waring	Abbvie, Chicago, IL, United States	jeff.waring@abbvie.com	Clinical Groups	**Gastroenterology Group**
Nizar Smaoui	Abbvie, Chicago, IL, United States	nizar.smaoui@abbvie.com	Clinical Groups	**Gastroenterology Group**
Fedik Rahimov	Abbvie, Chicago, IL, United States	fedik.rahimov@abbvie.com	Clinical Groups	**Gastroenterology Group**
Anne Lehtonen	Abbvie, Chicago, IL, United States	anne.lehtonen@abbvie.com	Clinical Groups	**Gastroenterology Group**
Tim Lu	Genentech, San Francisco, CA, United States	lut8@gene.com	Clinical Groups	**Gastroenterology Group**
Natalie Bowers	Genentech, San Francisco, CA, United States	bowersn1@gene.com	Clinical Groups	**Gastroenterology Group**
Rion Pendergrass	Genentech, San Francisco, CA, United States	penders2@gene.com	Clinical Groups	**Gastroenterology Group**
Linda McCarthy	GlaxoSmithKline, Brentford, United Kingdom	linda.c.mccarthy@gsk.com	Clinical Groups	**Gastroenterology Group**
Amy Hart	Janssen Research & Development, LLC, Spring House, PA, United States	ahart13@its.jnj.com	Clinical Groups	**Gastroenterology Group**
Meijian Guan	Janssen Research & Development, LLC, Spring House, PA, United States	mguan4@its.jnj.com	Clinical Groups	**Gastroenterology Group**
Jason Miller	Merck, Kenilworth, NJ, United States	jason.miller4@merck.com	Clinical Groups	**Gastroenterology Group**
Kirsi Kalpala	Pfizer, New York, NY, United States	kirsi.kalpala@pfizer.com	Clinical Groups	**Gastroenterology Group**
Melissa Miller	Pfizer, New York, NY, United States	melissa.r.miller@pfizer.com	Clinical Groups	**Gastroenterology Group**
Xinli Hu	Pfizer, New York, NY, United States	xinli.hu@pfizer.com	Clinical Groups	**Gastroenterology Group**
Kari Eklund	Hospital District of Helsinki and Uusimaa, Helsinki, Finland	kari.eklund@hus.fi	Clinical Groups	**Rheumatology Group**
Antti Palomäki	Hospital District of Southwest Finland, Turku, Finland	ajpalo@utu.fi	Clinical Groups	**Rheumatology Group**
Pia Isomäki	Pirkanmaa Hospital District, Tampere, Finland	pia.isomaki@pshp.fi	Clinical Groups	**Rheumatology Group**
Laura Pirilä	Hospital District of Southwest Finland, Turku, Finland	laura.pirila@fimnet.fi,laura.pirila@tyks.fi	Clinical Groups	**Rheumatology Group**
Oili Kaipiainen-Seppänen	Northern Savo Hospital District, Kuopio, Finland	oili.kaipiainen-seppanen@kuh.fi	Clinical Groups	**Rheumatology Group**
Johanna Huhtakangas	Northern Ostrobothnia Hospital District, Oulu, Finland	johanna.huhtakangas@kuh.fi	Clinical Groups	**Rheumatology Group**
Nina Mars	Institute for Molecular Medicine Finland (FIMM), HiLIFE, University of Helsinki, Helsinki, Finland	nina.mars@helsinki.fi	Clinical Groups	**Rheumatology Group**
Ali Abbasi	Abbvie, Chicago, IL, United States	ali.abbasi@abbvie.com	Clinical Groups	**Rheumatology Group**
Jeffrey Waring	Abbvie, Chicago, IL, United States	jeff.waring@abbvie.com	Clinical Groups	**Rheumatology Group**
Fedik Rahimov	Abbvie, Chicago, IL, United States	fedik.rahimov@abbvie.com	Clinical Groups	**Rheumatology Group**
Apinya Lertratanakul	Abbvie, Chicago, IL, United States	apinya.lertratanakul@abbvie.com	Clinical Groups	**Rheumatology Group**
Nizar Smaoui	Abbvie, Chicago, IL, United States	nizar.smaoui@abbvie.com	Clinical Groups	**Rheumatology Group**
Anne Lehtonen	Abbvie, Chicago, IL, United States	anne.lehtonen@abbvie.com	Clinical Groups	**Rheumatology Group**
Marla Hochfeld	Bristol Myers Squibb, New York, NY, United States	mhochfeld@celgene.com	Clinical Groups	**Rheumatology Group**
Natalie Bowers	Genentech, San Francisco, CA, United States	bowersn1@gene.com	Clinical Groups	**Rheumatology Group**
Rion Pendergrass	Genentech, San Francisco, CA, United States	penders2@gene.com	Clinical Groups	**Rheumatology Group**
Jorge Esparza Gordillo	GlaxoSmithKline, Brentford, United Kingdom	jorge.x.esparza-gordillo@gsk.com	Clinical Groups	**Rheumatology Group**
Kirsi Auro	GlaxoSmithKline, Espoo, Finland	kirsi.m.auro@gsk.com	Clinical Groups	**Rheumatology Group**
Dawn Waterworth	Janssen Research & Development, LLC, Spring House, PA, United States	dwaterwo@its.jnj.com	Clinical Groups	**Rheumatology Group**
Fabiana Farias	Merck, Kenilworth, NJ, United States	fabiana.farias@merck.com	Clinical Groups	**Rheumatology Group**
Kirsi Kalpala	Pfizer, New York, NY, United States	kirsi.kalpala@pfizer.com	Clinical Groups	**Rheumatology Group**
Nan Bing	Pfizer, New York, NY, United States	nan.bing@pfizer.com	Clinical Groups	**Rheumatology Group**
Xinli Hu	Pfizer, New York, NY, United States	xinli.hu@pfizer.com	Clinical Groups	**Rheumatology Group**
Tarja Laitinen	Pirkanmaa Hospital District, Tampere, Finland	tarja.laitinen@pshp.fi	Clinical Groups	**Pulmonology Group**
Margit Pelkonen	Northern Savo Hospital District, Kuopio, Finland	margit.pelkonen@kuh.fi	Clinical Groups	**Pulmonology Group**
Paula Kauppi	Hospital District of Helsinki and Uusimaa, Helsinki, Finland	paula.kauppi@hus.fi	Clinical Groups	**Pulmonology Group**
Hannu Kankaanranta	University of Gothenburg, Gothenburg, Sweden/ Seinäjoki Central Hospital, Seinäjoki, Finland/ Tampere University, Tampere, Finland	hannu.kankaanranta@tuni.fi	Clinical Groups	**Pulmonology Group**
Terttu Harju	Northern Ostrobothnia Hospital District, Oulu, Finland	terttu.harju@oulu.fi	Clinical Groups	**Pulmonology Group**
Riitta Lahesmaa	Hospital District of Southwest Finland, Turku, Finland	rilahes@utu.fi	Clinical Groups	**Pulmonology Group**
Nizar Smaoui	Abbvie, Chicago, IL, United States	nizar.smaoui@abbvie.com	Clinical Groups	**Pulmonology Group**
Glenda Lassi	Astra Zeneca, Cambridge, United Kingdom	glenda.lassi@astrazeneca.com	Clinical Groups	**Pulmonology Group**
Susan Eaton	Biogen, Cambridge, MA, United States	susan.eaton@biogen.com	Clinical Groups	**Pulmonology Group**
Hubert Chen	Genentech, San Francisco, CA, United States	chenh37@gene.com	Clinical Groups	**Pulmonology Group**
Rion Pendergrass	Genentech, San Francisco, CA, United States	penders2@gene.com	Clinical Groups	**Pulmonology Group**
Natalie Bowers	Genentech, San Francisco, CA, United States	bowersn1@gene.com	Clinical Groups	**Pulmonology Group**
Joanna Betts	GlaxoSmithKline, Brentford, United Kingdom	joanna.c.betts@gsk.com	Clinical Groups	**Pulmonology Group**
Kirsi Auro	GlaxoSmithKline, Espoo, Finland	kirsi.m.auro@gsk.com	Clinical Groups	**Pulmonology Group**
Rajashree Mishra	GlaxoSmithKline, Brentford, United Kingdom	rajashree.x.mishra@gsk.com	Clinical Groups	**Pulmonology Group**
Majd Mouded	Novartis, Basel, Switzerland	majd.mouded@novartis.com	Clinical Groups	**Pulmonology Group**
Debby Ngo	Novartis, Basel, Switzerland	debby.ngo@novartis.com	Clinical Groups	**Pulmonology Group**
Teemu Niiranen	Finnish Institute for Health and Welfare (THL), Helsinki, Finland	teemu.niiranen@thl.fi	Clinical Groups	**Cardiometabolic Diseases Group**
Felix Vaura	Finnish Institute for Health and Welfare (THL), Helsinki, Finland	fechva@utu.fi	Clinical Groups	**Cardiometabolic Diseases Group**
Veikko Salomaa	Finnish Institute for Health and Welfare (THL), Helsinki, Finland	veikko.salomaa@thl.fi	Clinical Groups	**Cardiometabolic Diseases Group**
Kaj Metsärinne	Hospital District of Southwest Finland, Turku, Finland	kaj.metsarinne@tyks.fi	Clinical Groups	**Cardiometabolic Diseases Group**
Jenni Aittokallio	Hospital District of Southwest Finland, Turku, Finland	jemato@utu.fi	Clinical Groups	**Cardiometabolic Diseases Group**
Mika Kähönen	Pirkanmaa Hospital District, Tampere, Finland	mika.kahonen@uta.fi	Clinical Groups	**Cardiometabolic Diseases Group**
Jussi Hernesniemi	Pirkanmaa Hospital District, Tampere, Finland	jussi.hernesniemi@tuni.fi	Clinical Groups	**Cardiometabolic Diseases Group**
Daniel Gordin	Hospital District of Helsinki and Uusimaa, Helsinki, Finland	daniel.gordin@hus.fi	Clinical Groups	**Cardiometabolic Diseases Group**
Juha Sinisalo	Hospital District of Helsinki and Uusimaa, Helsinki, Finland	juha.sinisalo@hus.fi	Clinical Groups	**Cardiometabolic Diseases Group**
Marja-Riitta Taskinen	Hospital District of Helsinki and Uusimaa, Helsinki, Finland	marja-riitta.taskinen@helsinki.fi	Clinical Groups	**Cardiometabolic Diseases Group**
Tiinamaija Tuomi	Hospital District of Helsinki and Uusimaa, Helsinki, Finland	tiinamaija.tuomi@hus.fi	Clinical Groups	**Cardiometabolic Diseases Group**
Timo Hiltunen	Hospital District of Helsinki and Uusimaa, Helsinki, Finland	timo.hiltunen@hus.fi	Clinical Groups	**Cardiometabolic Diseases Group**
Jari Laukkanen	Central Finland Health Care District, Jyväskylä, Finland	jari.laukkanen@ksshp.fi	Clinical Groups	**Cardiometabolic Diseases Group**
Amanda Elliott	Institute for Molecular Medicine Finland (FIMM), HiLIFE, University of Helsinki, Helsinki, Finland; Broad Institute, Cambridge, MA, USA and Massachusetts General Hospital, Boston, MA, USA	aelliott@broadinstitute.org	Clinical Groups	**Cardiometabolic Diseases Group**
Mary Pat Reeve	Institute for Molecular Medicine Finland (FIMM), HiLIFE, University of Helsinki, Helsinki, Finland	mary.reeve@helsinki.fi	Clinical Groups	**Cardiometabolic Diseases Group**
Sanni Ruotsalainen	Institute for Molecular Medicine Finland (FIMM), HiLIFE, University of Helsinki, Helsinki, Finland	sanni.ruotsalainen@helsinki.fi	Clinical Groups	**Cardiometabolic Diseases Group**
Benjamin Challis	Astra Zeneca, Cambridge, United Kingdom	benjamin.challis@astrazeneca.com	Clinical Groups	**Cardiometabolic Diseases Group**
Dirk Paul	Astra Zeneca, Cambridge, United Kingdom	dirk.paul@astrazeneca.com	Clinical Groups	**Cardiometabolic Diseases Group**
Julie Hunkapiller	Genentech, San Francisco, CA, United States	hunkapiller.julie@gene.com	Clinical Groups	**Cardiometabolic Diseases Group**
Natalie Bowers	Genentech, San Francisco, CA, United States	bowersn1@gene.com	Clinical Groups	**Cardiometabolic Diseases Group**
Rion Pendergrass	Genentech, San Francisco, CA, United States	penders2@gene.com	Clinical Groups	**Cardiometabolic Diseases Group**
Audrey Chu	GlaxoSmithKline, Brentford, United Kingdom	audrey.y.chu@gsk.com	Clinical Groups	**Cardiometabolic Diseases Group**
Kirsi Auro	GlaxoSmithKline, Espoo, Finland	kirsi.m.auro@gsk.com	Clinical Groups	**Cardiometabolic Diseases Group**
Dermot Reilly	Janssen Research & Development, LLC, Boston, MA, United States	dreill11@its.jnj.com	Clinical Groups	**Cardiometabolic Diseases Group**
Mike Mendelson	Novartis, Boston, MA, United States	mike.mendelson@novartis.com	Clinical Groups	**Cardiometabolic Diseases Group**
Jaakko Parkkinen	Pfizer, New York, NY, United States	jaakko.parkkinen@pfizer.com	Clinical Groups	**Cardiometabolic Diseases Group**
Melissa Miller	Pfizer, New York, NY, United States	melissa.r.miller@pfizer.com	Clinical Groups	**Cardiometabolic Diseases Group**
Tuomo Meretoja	Hospital District of Helsinki and Uusimaa, Helsinki, Finland	tuomo.meretoja@hus.fi	Clinical Groups	**Oncology Group**
Heikki Joensuu	Hospital District of Helsinki and Uusimaa, Helsinki, Finland	heikki.joensuu@hus.fi	Clinical Groups	**Oncology Group**
Olli Carpén	Hospital District of Helsinki and Uusimaa, Helsinki, Finland	olli.carpen@helsinki.fi	Clinical Groups	**Oncology Group**
Johanna Mattson	Hospital District of Helsinki and Uusimaa, Helsinki, Finland	johanna.mattson@hus.fi	Clinical Groups	**Oncology Group**
Eveliina Salminen	Hospital District of Helsinki and Uusimaa, Helsinki, Finland	eveliina.e.salminen@hus.fi	Clinical Groups	**Oncology Group**
Annika Auranen	Pirkanmaa Hospital District , Tampere, Finland	anaura@utu.fi	Clinical Groups	**Oncology Group**
Peeter Karihtala	Northern Ostrobothnia Hospital District, Oulu, Finland	peeter.karihtala@oulu.fi	Clinical Groups	**Oncology Group**

Päivi Auvinen	Northern Savo Hospital District, Kuopio, Finland	paivi.auvinen@kuh.fi	Clinical Groups	Oncology Group
Klaus Elenius	Hospital District of Southwest Finland, Turku, Finland	klaus.elenius@utu.fi	Clinical Groups	Oncology Group
Johanna Schleutker	Hospital District of Southwest Finland, Turku, Finland	johanna.schleutker@utu.fi	Clinical Groups	Oncology Group
Esa Pitkänen	Institute for Molecular Medicine Finland (FIMM), HiLIFE, University of Helsinki, Helsinki, Finland	esa.pitkanen@helsinki.fi	Clinical Groups	Oncology Group
Nina Mars	Institute for Molecular Medicine Finland (FIMM), HiLIFE, University of Helsinki, Helsinki, Finland	nina.mars@helsinki.fi	Clinical Groups	Oncology Group
Mark Daly	Institute for Molecular Medicine Finland (FIMM), HiLIFE, University of Helsinki, Helsinki, Finland; Broad Institute of MIT and Harvard; Massachusetts General Hospital	mark.daly@helsinki.fi	Clinical Groups	Oncology Group
Relja Popovic	Abbvie, Chicago, IL, United States	relja.popovic@abbvie.com	Clinical Groups	Oncology Group
Jeffrey Waring	Abbvie, Chicago, IL, United States	jeff.waring@abbvie.com	Clinical Groups	Oncology Group
Bridget Riley-Gillis	Abbvie, Chicago, IL, United States	bridget.rileygillis@abbvie.com	Clinical Groups	Oncology Group
Anne Lehtonen	Abbvie, Chicago, IL, United States	anne.lehtonen@abbvie.com	Clinical Groups	Oncology Group
Jennifer Schutzman	Genentech, San Francisco, CA, United States	schutzman.jennifer@gene.com	Clinical Groups	Oncology Group
Julie Hunkapiller	Genentech, San Francisco, CA, United States	hunkapiller.julie@gene.com	Clinical Groups	Oncology Group
Natalie Bowers	Genentech, San Francisco, CA, United States	bowersn1@gene.com	Clinical Groups	Oncology Group
Rion Pendergrass	Genentech, San Francisco, CA, United States	penders2@gene.com	Clinical Groups	Oncology Group
Diptee Kulkarni	GlaxoSmithKline, Brentford, United Kingdom	diptee.a.kulkarni@gsk.com	Clinical Groups	Oncology Group
Kirsi Auro	GlaxoSmithKline, Espoo, Finland	kirsi.m.auro@gsk.com	Clinical Groups	Oncology Group
Alessandro Porello	Janssen Research & Development, LLC, Spring House, PA, United States	APorrell@ITS.JNJ.com	Clinical Groups	Oncology Group
Andrey Loboda	Merck, Kenilworth, NJ, United States	andrey_loboda@merck.com	Clinical Groups	Oncology Group
Heli Lehtonen	Pfizer, New York, NY, United States	heli.lehtonen@pfizer.com	Clinical Groups	Oncology Group
Stefan McDonough	Pfizer, New York, NY, United States	stefan.McDonough@pfizer.com	Clinical Groups	Oncology Group
Sauli Vuoti	Janssen-Cilag Oy, Espoo, Finland	svuoti@its.jnj.com	Clinical Groups	Oncology Group
Kai Kaarniranta	Northern Savo Hospital District, Kuopio, Finland	kai.kaarniranta@uef.fi	Clinical Groups	Opthalmology Group
Joni A Turunen	Helsinki University Hospital and University of Helsinki, Helsinki, Finland; Eye Genetics Group, Folkhälsan Research Center, Helsinki, Finland	joni.turunen@helsinki.fi	Clinical Groups	Opthalmology Group
Terhi Ollila	Hospital District of Helsinki and Uusimaa, Helsinki, Finland	terhi.ollila@hus.fi	Clinical Groups	Opthalmology Group
Hannu Uusitalo	Pirkanmaa Hospital District, Tampere, Finland	hannu.uusitalo@tuni.fi	Clinical Groups	Opthalmology Group
Juha Karjalainen	Institute for Molecular Medicine Finland (FIMM), HiLIFE, University of Helsinki, Helsinki, Finland	juha.karjalainen@helsinki.fi	Clinical Groups	Opthalmology Group
Esa Pitkänen	Institute for Molecular Medicine Finland (FIMM), HiLIFE, University of Helsinki, Helsinki, Finland	esa.pitkanen@helsinki.fi	Clinical Groups	Opthalmology Group
Mengzhen Liu	Abbvie, Chicago, IL, United States	mengzhen.liu@abbvie.com	Clinical Groups	Opthalmology Group
Heiko Runz	Biogen, Cambridge, MA, United States	heiko.runz@biogen.com	Clinical Groups	Opthalmology Group
Stephanie Loomis	Biogen, Cambridge, MA, United States	stephanie.loomis@biogen.com	Clinical Groups	Opthalmology Group
Erich Strauss	Genentech, San Francisco, CA, United States	strauss.erich@gene.com	Clinical Groups	Opthalmology Group
Natalie Bowers	Genentech, San Francisco, CA, United States	bowersn1@gene.com	Clinical Groups	Opthalmology Group
Hao Chen	Genentech, San Francisco, CA, United States	haoc@gene.com	Clinical Groups	Opthalmology Group
Rion Pendergrass	Genentech, San Francisco, CA, United States	penders2@gene.com	Clinical Groups	Opthalmology Group
Kaisa Tasanen	Northern Ostrobothnia Hospital District, Oulu, Finland	kaisa.tasanen-maatta@oulu.fi	Clinical Groups	Dermatology Group
Laura Huilaja	Northern Ostrobothnia Hospital District, Oulu, Finland	laura.huilaja@oulu.fi	Clinical Groups	Dermatology Group
Katariina Hannula-Jouppi	Hospital District of Helsinki and Uusimaa, Helsinki, Finland	katariina.hannula-jouppi@hus.fi	Clinical Groups	Dermatology Group
Teea Salmi	Pirkanmaa Hospital District, Tampere, Finland	teea.salmi@pshp.fi	Clinical Groups	Dermatology Group
Sirkku Peltonen	Hospital District of Southwest Finland, Turku, Finland	sipelto@utu.fi	Clinical Groups	Dermatology Group
Leena Koulu	Hospital District of Southwest Finland, Turku, Finland	leena.koulu@tyks.fi	Clinical Groups	Dermatology Group
Nizar Smaoui	Abbvie, Chicago, IL, United States	nizar.smaoui@abbvie.com	Clinical Groups	Dermatology Group
Fedik Rahimov	Abbvie, Chicago, IL, United States	fedik.rahimov@abbvie.com	Clinical Groups	Dermatology Group
Anne Lehtonen	Abbvie, Chicago, IL, United States	anne.lehtonen@abbvie.com	Clinical Groups	Dermatology Group
David Choy	Genentech, San Francisco, CA, United States	choy.david@gene.com	Clinical Groups	Dermatology Group
Rion Pendergrass	Genentech, San Francisco, CA, United States	penders2@gene.com	Clinical Groups	Dermatology Group
Dawn Waterworth	Janssen Research & Development, LLC, Spring House, PA, United States	dwaterwo@its.jnj.com	Clinical Groups	Dermatology Group
Kirsi Kalpala	Pfizer, New York, NY, United States	kirsi.kalpala@pfizer.com	Clinical Groups	Dermatology Group
Ying Wu	Pfizer, New York, NY, United States	ying.wu3@pfizer.com	Clinical Groups	Dermatology Group
Pirkko Pussinen	Hospital District of Helsinki and Uusimaa, Helsinki, Finland	pirkko.pussinen@helsinki.fi	Clinical Groups	Odontology Group
Aino Salminen	Hospital District of Helsinki and Uusimaa, Helsinki, Finland	aino.m.salminen@helsinki.fi	Clinical Groups	Odontology Group
Tuula Salo	Hospital District of Helsinki and Uusimaa, Helsinki, Finland	tuula.salo@helsinki.fi	Clinical Groups	Odontology Group
David Rice	Hospital District of Helsinki and Uusimaa, Helsinki, Finland	david.rice@helsinki.fi	Clinical Groups	Odontology Group
Pekka Nieminen	Hospital District of Helsinki and Uusimaa, Helsinki, Finland	pekka.nieminen@helsinki.fi	Clinical Groups	Odontology Group
Ulla Palotie	Hospital District of Helsinki and Uusimaa, Helsinki, Finland	ulla.palotie@helsinki.fi	Clinical Groups	Odontology Group
Maria Siponen	Northern Savo Hospital District, Kuopio, Finland	maria.siponen@uef.fi	Clinical Groups	Odontology Group
Liisa Suominen	Northern Savo Hospital District, Kuopio, Finland	liisa.suominen@uef.fi	Clinical Groups	Odontology Group
Päivi Mäntylä	Northern Savo Hospital District, Kuopio, Finland	paivi.mantyla@uef.fi	Clinical Groups	Odontology Group
Ulvi Gursoy	Hospital District of Southwest Finland, Turku, Finland	ulvi.gursoy@utu.fi	Clinical Groups	Odontology Group
Vuokko Anttonen	Northern Ostrobothnia Hospital District, Oulu, Finland	vuokko.anttonen@oulu.fi	Clinical Groups	Odontology Group
Kirsi Sipilä	Research Unit of Oral Health Sciences Faculty of Medicine, University of Oulu, Oulu, Finland; Medical Research Center, Oulu, Oulu University Hospital and University of Oulu, Oulu, Finland	kirsi.sipila@oulu.fi	Clinical Groups	Odontology Group
Rion Pendergrass	Genentech, San Francisco, CA, United States	pendergrass.sarah@gene.com	Clinical Groups	Odontology Group
Hannele Laivuori	Institute for Molecular Medicine Finland (FIMM), HiLIFE, University of Helsinki, Helsinki, Finland	hannele.laivuori@helsinki.fi	Clinical Groups	Women's Health and Reproduction Group
Venla Kurra	Pirkanmaa Hospital District, Tampere, Finland	venla.kurra@tuni.fi	Clinical Groups	Women's Health and Reproduction Group
Laura Kotaniemi-Talonen	Pirkanmaa Hospital District, Tampere, Finland	laura.kotaniemi-talonen@tuni.fi	Clinical Groups	Women's Health and Reproduction Group
Oskari Heikinheimo	Hospital District of Helsinki and Uusimaa, Helsinki, Finland	oskari.heikinheimo@helsinki.fi	Clinical Groups	Women's Health and Reproduction Group
Ilkka Kalliala	Hospital District of Helsinki and Uusimaa, Helsinki, Finland	ilkka.kalliala@hus.fi	Clinical Groups	Women's Health and Reproduction Group
Lauri Aaltonen	Hospital District of Helsinki and Uusimaa, Helsinki, Finland	lauri.aaltonen@helsinki.fi	Clinical Groups	Women's Health and Reproduction Group
Varpu Jokimaa	Hospital District of Southwest Finland, Turku, Finland	varpu.jokimaa@utu.fi	Clinical Groups	Women's Health and Reproduction Group
Johannes Kettunen	Northern Ostrobothnia Hospital District, Oulu, Finland	Johannes.Kettunen@oulu.fi	Clinical Groups	Women's Health and Reproduction Group
Marja Vääräsmäki	Northern Ostrobothnia Hospital District, Oulu, Finland	marja.vaarasmaki@oulu.fi	Clinical Groups	Women's Health and Reproduction Group
Outi Uimari	Northern Ostrobothnia Hospital District, Oulu, Finland	outi.uimari@oulu.fi	Clinical Groups	Women's Health and Reproduction Group
Laure Morin-Papunen	Northern Ostrobothnia Hospital District, Oulu, Finland	lmp@cc.oulu.fi	Clinical Groups	Women's Health and Reproduction Group
Maarit Niinimäki	Northern Ostrobothnia Hospital District, Oulu, Finland	maarit.niinimaki@oulu.fi	Clinical Groups	Women's Health and Reproduction Group
Terhi Piltonen	Northern Ostrobothnia Hospital District, Oulu, Finland	terhi.piltonen@oulu.fi	Clinical Groups	Women's Health and Reproduction Group
Katja Kivinen	Institute for Molecular Medicine Finland (FIMM), HiLIFE, University of Helsinki, Helsinki, Finland	katja.kivinen@helsinki.fi	Clinical Groups	Women's Health and Reproduction Group
Elisabeth Widen	Institute for Molecular Medicine Finland (FIMM), HiLIFE, University of Helsinki, Helsinki, Finland	elisabeth.widen@helsinki.fi	Clinical Groups	Women's Health and Reproduction Group
Taru Tukiainen	Institute for Molecular Medicine Finland (FIMM), HiLIFE, University of Helsinki, Helsinki, Finland	taru.tukiainen@helsinki.fi	Clinical Groups	Women's Health and Reproduction Group
Mary Pat Reeve	Institute for Molecular Medicine Finland (FIMM), HiLIFE, University of Helsinki, Helsinki, Finland	mary.reeve@helsinki.fi	Clinical Groups	Women's Health and Reproduction Group
Mark Daly	Institute for Molecular Medicine Finland (FIMM), HiLIFE, University of Helsinki, Helsinki, Finland; Broad Institute of MIT and Harvard; Massachusetts General Hospital	mark.daly@helsinki.fi	Clinical Groups	Women's Health and Reproduction Group
Niko Välimäki	University of Helsinki, Helsinki, Finland	niko.valimaki@helsinki.fi	Clinical Groups	Women's Health and Reproduction Group
Eija Laakkonen	University of Jyväskylä, Jyväskylä, Finland	eija.k.laakkonen@jyu.fi	Clinical Groups	Women's Health and Reproduction Group
Jaakko Tyrmi	University of Oulu, Oulu, Finland / University of Tampere, Tampere, Finland	jaakko.tyrmi@oulu.fi	Clinical Groups	Women's Health and Reproduction Group
Heidi Silven	University of Oulu, Oulu, Finland	heidi.silven@student.oulu.fi	Clinical Groups	Women's Health and Reproduction Group
Eeva Sliz	University of Oulu, Oulu, Finland	eeva.sliz@oulu.fi	Clinical Groups	Women's Health and Reproduction Group
Riikka Arffman	University of Oulu, Oulu, Finland	riikka.arffman@oulu.fi	Clinical Groups	Women's Health and Reproduction Group
Susanna Savukoski	University of Oulu, Oulu, Finland	susanna.savukoski@oulu.fi	Clinical Groups	Women's Health and Reproduction Group
Triin Laisk	Estonian biobank, Tartu, Estonia	triin.laisk@ut.ee	Clinical Groups	Women's Health and Reproduction Group
Natalia Pujol	Estonian biobank, Tartu, Estonia	natalia.pujolgualdo@oulu.fi	Clinical Groups	Women's Health and Reproduction Group
Mengzhen Liu	Abbvie, Chicago, IL, United States	mengzhen.liu@abbvie.com	Clinical Groups	Women's Health and Reproduction Group
Bridget Riley-Gillis	Abbvie, Chicago, IL, United States	bridget.rileygillis@abbvie.com	Clinical Groups	Women's Health and Reproduction Group
Rion Pendergrass	Genentech, San Francisco, CA, United States	penders2@gene.com	Clinical Groups	Women's Health and Reproduction Group
Janet Kumar	GlaxoSmithKline, Collegeville, PA, United States	janet.x.kumar@gsk.com	Clinical Groups	Women's Health and Reproduction Group
Kirsi Auro	GlaxoSmithKline, Espoo, Finland	kirsi.m.auro@gsk.com	Clinical Groups	Women's Health and Reproduction Group
Iiris Hovatta	University of Helsinki, Finland	iiris.hovatta@helsinki.fi	Clinical Groups	Depression group
Chia-Yen Chen	Biogen, Cambridge, MA, United States	chiayen.chen@biogen.com	Clinical Groups	Depression group
Erkki Isometsä	Hospital District of Helsinki and Uusimaa, Helsinki, Finland	erkki.isometsa@hus.fi	Clinical Groups	Depression group
Hanna Ollila	Institute for Molecular Medicine Finland (FIMM), HiLIFE, University of Helsinki, Helsinki, Finland	hanna.m.ollila@helsinki.fi	Clinical Groups	Depression group
Jaana Suvisaari	Finnish Institute for Health and Welfare (THL), Helsinki, Finland	jaana.suvisaari@thl.fi	Clinical Groups	Depression group
Thomas Damm Als	Aarhus University, Denmark	tda@biomed.au.dk	Clinical Groups	Depression group
Antti Mäkitie	Department of Otorhinolaryngology - Head and Neck Surgery, University of Helsinki and Helsinki University Hospital, Helsinki, Finland	antti.makitie@helsinki.fi	Clinical Groups	ENT (ear, nose and throath) Group
Argyro Bizaki-Vallaskangas	Pirkanmaa Hospital District, Tampere, Finland	argyro.bizaki-vallaskangas@tuni.fi	Clinical Groups	ENT (ear, nose and throath) Group
Sanna Toppila-Salmi	University of Helsinki, Finland	sanna.salmi@helsinki.fi	Clinical Groups	ENT (ear, nose and throath) Group
Tytti Willberg	Hospital District of Southwest Finland, Turku, Finland	tytti.willberg@tyks.fi	Clinical Groups	ENT (ear, nose and throath) Group
Elmo Saarentaus	Institute for Molecular Medicine Finland (FIMM), HiLIFE, University of Helsinki, Helsinki, Finland	elmo.saarentaus@helsinki.fi	Clinical Groups	ENT (ear, nose and throath) Group
Antti Aarnisalo	Hospital District of Helsinki and Uusimaa, Helsinki, Finland	antti.aarnisalo@hus.fi	Clinical Groups	ENT (ear, nose and throath) Group
Eveliina Salminen	Hospital District of Helsinki and Uusimaa, Helsinki, Finland	eveliina.e.salminen@hus.fi	Clinical Groups	ENT (ear, nose and throath) Group
Elisa Rahikkala	Northern Ostrobothnia Hospital District, Oulu, Finland	elisa.rahikkala@ppshp.fi	Clinical Groups	ENT (ear, nose and throath) Group

Johannes Kettunen	Northern Ostrobothnia Hospital District, Oulu, Finland	johannes.kettunen@oulu.fi	Clinical Groups	**ENT (ear, nose and throath) Group**
Kristiina Aittomäki	Department of Medical Genetics, Helsinki University Central Hospital, Helsinki, Finland	kristiina.aittomaki@helsinki.fi	Clinical Groups	**POI (premature ovarian failure) Group**
Fredrik Åberg	Transplantation and Liver Surgery Clinic, Helsinki University Hospital, Helsinki University, Helsinki, Finland	fredrik.aberg@helsinki.fi	Clinical Groups	**LiverScore Group**
Mitja Kurki	Institute for Molecular Medicine Finland (FIMM), HiLIFE, University of Helsinki, Helsinki, Finland; Broad Institute, Cambridge, MA, United States	mkurki@broadinstitute.org	FinnGen Analysis working gro	FinnGen Analysis working group
Samuli Ripatti	Institute for Molecular Medicine Finland (FIMM), HiLIFE, University of Helsinki, Helsinki, Finland	samuli.ripatti@helsinki.fi	FinnGen Analysis working gro	FinnGen Analysis working group
Mark Daly	Institute for Molecular Medicine, Finland (FIMM), HiLIFE, University of Helsinki, Helsinki, Finland; Broad Institute of MIT and Harvard; Massachusetts General Hospital	mark.daly@helsinki.fi	FinnGen Analysis working gro	FinnGen Analysis working group
Juha Karjalainen	Institute for Molecular Medicine Finland (FIMM), HiLIFE, University of Helsinki, Helsinki, Finland	juha.karjalainen@helsinki.fi	FinnGen Analysis working gro	FinnGen Analysis working group
Aki Havulinna	Institute for Molecular Medicine Finland (FIMM), HiLIFE, University of Helsinki, Helsinki, Finland; Finnish Institute for Health and Welfare (THL), Helsinki, Finland	aki.havulinna@helsinki.fi	FinnGen Analysis working gro	FinnGen Analysis working group
Juha Mehtonen	Institute for Molecular Medicine Finland (FIMM), HiLIFE, University of Helsinki, Helsinki, Finland	juha.mehtonen@helsinki.fi	FinnGen Analysis working gro	FinnGen Analysis working group
Priit Palta	Institute for Molecular Medicine Finland (FIMM), HiLIFE, University of Helsinki, Helsinki, Finland	priit.palta@helsinki.fi	FinnGen Analysis working gro	FinnGen Analysis working group
Shabbeer Hassan	Institute for Molecular Medicine Finland (FIMM), HiLIFE, University of Helsinki, Helsinki, Finland	shabbeer.hassan@helsinki.fi	FinnGen Analysis working gro	FinnGen Analysis working group
Pietro Della Briotta Parolo	Institute for Molecular Medicine Finland (FIMM), HiLIFE, University of Helsinki, Helsinki, Finland	pietro.dellabriottaparolo@helsinki.fi	FinnGen Analysis working gro	FinnGen Analysis working group
Wei Zhou	Broad Institute, Cambridge, MA, United States	wzhou@broadinstitute.org	FinnGen Analysis working gro	FinnGen Analysis working group
Mutaamba Maasha	Broad Institute, Cambridge, MA, United States	mmaasha@broadinstitute.org	FinnGen Analysis working gro	FinnGen Analysis working group
Shabbeer Hassan	Institute for Molecular Medicine Finland (FIMM), HiLIFE, University of Helsinki, Helsinki, Finland	shabbeer.hassan@helsinki.fi	FinnGen Analysis working gro	FinnGen Analysis working group
Susanna Lemmelä	Institute for Molecular Medicine Finland (FIMM), HiLIFE, University of Helsinki, Helsinki, Finland	susanna.lemmela@helsinki.fi	FinnGen Analysis working gro	FinnGen Analysis working group
Manuel Rivas	University of Stanford, Stanford, CA, United States	mrivas@stanford.edu	FinnGen Analysis working gro	FinnGen Analysis working group
Mari E. Niemi	Institute for Molecular Medicine Finland (FIMM), HiLIFE, University of Helsinki, Helsinki, Finland	mari.e.niemi@helsinki.fi	FinnGen Analysis working gro	FinnGen Analysis working group
Aarno Palotie	Institute for Molecular Medicine Finland (FIMM), HiLIFE, University of Helsinki, Helsinki, Finland	aarno.palotie@helsinki.fi	FinnGen Analysis working gro	FinnGen Analysis working group
Aoxing Liu	Institute for Molecular Medicine Finland (FIMM), HiLIFE, University of Helsinki, Helsinki, Finland	aoxing.liu@helsinki.fi	FinnGen Analysis working gro	FinnGen Analysis working group
Arto Lehisto	Institute for Molecular Medicine Finland (FIMM), HiLIFE, University of Helsinki, Helsinki, Finland	arto.lehisto@helsinki.fi	FinnGen Analysis working gro	FinnGen Analysis working group
Andrea Ganna	Institute for Molecular Medicine Finland (FIMM), HiLIFE, University of Helsinki, Helsinki, Finland	aganna@broadinstitute.org	FinnGen Analysis working gro	FinnGen Analysis working group
Vincent Llorens	Institute for Molecular Medicine Finland (FIMM), HiLIFE, University of Helsinki, Helsinki, Finland	vincent.llorens@helsinki.fi	FinnGen Analysis working gro	FinnGen Analysis working group
Hannele Laivuori	Institute for Molecular Medicine Finland (FIMM), HiLIFE, University of Helsinki, Helsinki, Finland	hannele.laivuori@helsinki.fi	FinnGen Analysis working gro	FinnGen Analysis working group
Taru Tukiainen	Institute for Molecular Medicine Finland (FIMM), HiLIFE, University of Helsinki, Helsinki, Finland	taru.tukiainen@helsinki.fi	FinnGen Analysis working gro	FinnGen Analysis working group
Mary Pat Reeve	Institute for Molecular Medicine Finland (FIMM), HiLIFE, University of Helsinki, Helsinki, Finland	mary.reeve@helsinki.fi	FinnGen Analysis working gro	FinnGen Analysis working group
Henrike Heyne	Institute for Molecular Medicine Finland (FIMM), HiLIFE, University of Helsinki, Helsinki, Finland	hheyne@broadinstitute.org	FinnGen Analysis working gro	FinnGen Analysis working group
Nina Mars	Institute for Molecular Medicine Finland (FIMM), HiLIFE, University of Helsinki, Helsinki, Finland	nina.mars@helsinki.fi	FinnGen Analysis working gro	FinnGen Analysis working group
Joel Rämö	Institute for Molecular Medicine Finland (FIMM), HiLIFE, University of Helsinki, Helsinki, Finland	joel.ramo@helsinki.fi	FinnGen Analysis working gro	FinnGen Analysis working group
Elmo Saarentaus	Institute for Molecular Medicine Finland (FIMM), HiLIFE, University of Helsinki, Helsinki, Finland	elmo.saarentaus@helsinki.fi	FinnGen Analysis working gro	FinnGen Analysis working group
Hanna Ollila	Institute for Molecular Medicine Finland (FIMM), HiLIFE, University of Helsinki, Helsinki, Finland	hanna.m.ollila@helsinki.fi	FinnGen Analysis working gro	FinnGen Analysis working group
Rodos Rodosthenous	Institute for Molecular Medicine Finland (FIMM), HiLIFE, University of Helsinki, Helsinki, Finland	rodos.rodosthenous@helsinki.fi	FinnGen Analysis working gro	FinnGen Analysis working group
Satu Strausz	Institute for Molecular Medicine Finland (FIMM), HiLIFE, University of Helsinki, Helsinki, Finland	satu.strausz@helsinki.fi	FinnGen Analysis working gro	FinnGen Analysis working group
Tuula Palotie	University of Helsinki and Hospital District of Helsinki and Uusimaa, Helsinki, Finland	tuula.palotie@helsinki.fi	FinnGen Analysis working gro	FinnGen Analysis working group
Kimmo Palin	University of Helsinki, Helsinki, Finland	kimmo.palin@helsinki.fi	FinnGen Analysis working gro	FinnGen Analysis working group
Javier Garcia-Tabuenca	University of Tampere, Tampere, Finland	javier.graciatabuenca@tuni.fi	FinnGen Analysis working gro	FinnGen Analysis working group
Harri Siirtola	University of Tampere, Tampere, Finland	harri.siirtola@tuni.fi	FinnGen Analysis working gro	FinnGen Analysis working group
Tuomo Kiiskinen	Institute for Molecular Medicine Finland (FIMM), HiLIFE, University of Helsinki, Helsinki, Finland	tuomo.kiiskinen@helsinki.fi	FinnGen Analysis working gro	FinnGen Analysis working group
Jiwoo Lee	Institute for Molecular Medicine Finland (FIMM), HiLIFE, University of Helsinki, Helsinki, Finland; Broad Institute, Cambridge, MA, United States	jiwoo.lee@helsinki.fi	FinnGen Analysis working gro	FinnGen Analysis working group
Kristin Tsuo	Institute for Molecular Medicine Finland (FIMM), HiLIFE, University of Helsinki, Helsinki, Finland; Broad Institute, Cambridge, MA, United States	kristintsuo@fas.harvard.edu	FinnGen Analysis working gro	FinnGen Analysis working group
Amanda Elliott	Institute for Molecular Medicine Finland (FIMM), HiLIFE, University of Helsinki, Helsinki, Finland; Broad Institute, Cambridge, MA, USA and Massachusetts General Hospital, Boston, MA, USA	aelliott@broadinstitute.org	FinnGen Analysis working gro	FinnGen Analysis working group
Kati Kristiansson	THL Biobank / Finnish Institute for Health and Welfare (THL), Helsinki, Finland	kati.kristiansson@thl.fi	FinnGen Analysis working gro	FinnGen Analysis working group
Mikko Arvas	Finnish Red Cross Blood Service / Finnish Hematology Registry and Clinical Biobank, Helsinki, Finland	mikko.arvas@veripalvelu.fi	FinnGen Analysis working gro	FinnGen Analysis working group
Kati Hyvärinen	Finnish Red Cross Blood Service, Helsinki, Finland	kati.hyvarinen@veripalvelu.fi	FinnGen Analysis working gro	FinnGen Analysis working group
Jarmo Ritari	Finnish Red Cross Blood Service, Helsinki, Finland	jarmo.ritari@veripalvelu.fi	FinnGen Analysis working gro	FinnGen Analysis working group
Olli Carpén	Helsinki Biobank / Helsinki University and Hospital District of Helsinki and Uusimaa, Helsinki	olli.carpen@helsinki.fi	FinnGen Analysis working gro	FinnGen Analysis working group
Johannes Kettunen	Northern Finland Biobank Borealis / University of Oulu / Northern Ostrobothnia Hospital District, Oulu, Finland	johannes.kettunen@oulu.fi	FinnGen Analysis working gro	FinnGen Analysis working group
Katri Pylkäs	University of Oulu, Oulu, Finland	katri.pylkas@oulu.fi	FinnGen Analysis working gro	FinnGen Analysis working group
Eeva Sliz	University of Oulu, Oulu, Finland	eeva.sliz@oulu.fi	FinnGen Analysis working gro	FinnGen Analysis working group
Minna Karjalainen	University of Oulu, Oulu, Finland	minna.k.karjalainen@oulu.fi	FinnGen Analysis working gro	FinnGen Analysis working group
Tuomo Mantere	Northern Finland Biobank Borealis / University of Oulu / Northern Ostrobothnia Hospital District, Oulu, Finland	tuomo.mantere@oulu.fi	FinnGen Analysis working gro	FinnGen Analysis working group
Eeva Kangasniemi	Finnish Clinical Biobank Tampere / University of Tampere / Pirkanmaa Hospital District, Tampere, Finland	eeva.kangasniemi@pshp.fi	FinnGen Analysis working gro	FinnGen Analysis working group
Sami Heikkinen	University of Eastern Finland, Kuopio, Finland	sami.heikkinen@uef.fi	FinnGen Analysis working gro	FinnGen Analysis working group
Arto Mannermaa	Biobank of Eastern Finland / University of Eastern Finland / Northern Savo Hospital District, Kuopio, Finland	arto.mannermaa@uef.fi	FinnGen Analysis working gro	FinnGen Analysis working group
Eija Laakkonen	University of Jyväskylä, Jyväskylä, Finland	eija.k.laakkonen@jyu.fi	FinnGen Analysis working gro	FinnGen Analysis working group
Nina Pitkänen	Auria Biobank / University of Turku / Hospital District of Southwest Finland, Turku, Finland	Niina.Pitkanen@tyks.fi	FinnGen Analysis working gro	FinnGen Analysis working group
Samuel Lessard	Translational Sciences, Sanofi R&D, Framingham, MA, USA	samuel.lessard@sanofi.com	FinnGen Analysis working gro	FinnGen Analysis working group
Clément Chatelain	Translational Sciences, Sanofi R&D, Framingham, MA, USA	clement.chatelain@sanofi.com	FinnGen Analysis working gro	FinnGen Analysis working group
Perttu Terho	Auria Biobank / University of Turku / Hospital District of Southwest Finland, Turku, Finland	perttu.terho@tyks.fi	Biobank directors	Biobank directors
Sirpa Soini	THL Biobank / Finnish Institute for Health and Welfare (THL), Helsinki, Finland	sirpa.soini@thl.fi	Biobank directors	Biobank directors
Jukka Partanen	Finnish Red Cross Blood Service / Finnish Hematology Registry and Clinical Biobank, Helsinki, Finland	jukka.partanen@veripalvelu.fi	Biobank directors	Biobank directors
Eero Punkka	Helsinki Biobank / Helsinki University and Hospital District of Helsinki and Uusimaa, Helsinki	eero.punkka@hus.fi	Biobank directors	Biobank directors
Raisa Serpi	Northern Finland Biobank Borealis / University of Oulu / Northern Ostrobothnia Hospital District, Oulu, Finland	raisa.serpi@ppshp.fi	Biobank directors	Biobank directors
Sanna Siltanen	Finnish Clinical Biobank Tampere / University of Tampere / Pirkanmaa Hospital District, Tampere, Finland	sanna.siltanen@pshp.fi	Biobank directors	Biobank directors
Veli-Matti Kosma	Biobank of Eastern Finland / University of Eastern Finland / Northern Savo Hospital District, Kuopio, Finland	veli-matti.kosma@uef.fi	Biobank directors	Biobank directors
Teijo Kuopio	Central Finland Biobank / University of Jyväskylä / Central Finland Health Care District, Jyväskylä, Finland	teijo.kuopio@ksshp.fi	Biobank directors	Biobank directors
Anu Jalanko	Institute for Molecular Medicine Finland (FIMM), HiLIFE, University of Helsinki, Helsinki, Finland	anu.jalanko@helsinki.fi	FinnGen Teams	**Administration**
Huei-Yi Shen	Institute for Molecular Medicine Finland (FIMM), HiLIFE, University of Helsinki, Helsinki, Finland	huei-yi.shen@helsinki.fi	FinnGen Teams	**Administration**
Risto Kajanne	Institute for Molecular Medicine Finland (FIMM), HiLIFE, University of Helsinki, Helsinki, Finland	risto.kajanne@helsinki.fi	FinnGen Teams	**Administration**
Mervi Aavikko	Institute for Molecular Medicine Finland (FIMM), HiLIFE, University of Helsinki, Helsinki, Finland	mervi.aavikko@helsinki.fi	FinnGen Teams	**Administration**
Henna Palin	Finnish Clinical Biobank Tampere / University of Tampere / Pirkanmaa Hospital District, Tampere, Finland	henna.palin@pshp.fi	FinnGen Teams	**Administration**

Malla-Maria Linna	Helsinki Biobank / Helsinki University and Hospital District of Helsinki and Uusimaa, Helsinki	malla-maria.linna@hus.fi	FinnGen Teams	**Administration**
Mitja Kurki	Institute for Molecular Medicine Finland (FIMM), HiLIFE, University of Helsinki, Helsinki, Finland; Broad Institute, Cambridge, MA, United States	mkurki@broadinstitute.org	FinnGen Teams	**Analysis**
Juha Karjalainen	Institute for Molecular Medicine Finland (FIMM), HiLIFE, University of Helsinki, Helsinki, Finland	juha.karjalainen@helsinki.fi	FinnGen Teams	**Analysis**
Pietro Della Briotta Parolo	Institute for Molecular Medicine Finland (FIMM), HiLIFE, University of Helsinki, Helsinki, Finland	pietro.dellabriottaparolo@helsinki.fi	FinnGen Teams	**Analysis**
Arto Lehisto	Institute for Molecular Medicine Finland (FIMM), HiLIFE, University of Helsinki, Helsinki, Finland	arto.lehisto@helsinki.fi	FinnGen Teams	**Analysis**
Juha Mehtonen	Institute for Molecular Medicine Finland (FIMM), HiLIFE, University of Helsinki, Helsinki, Finland	juha.mehtonen@helsinki.fi	FinnGen Teams	**Analysis**
Wei Zhou	Broad Institute, Cambridge, MA, United States	wzhou@broadinstitute.org	FinnGen Teams	**Analysis**
Masahiro Kanai	Broad Institute, Cambridge, MA, United States	mkanai@broadinstitute.org	FinnGen Teams	**Analysis**
Mutaamba Maasha	Broad Institute, Cambridge, MA, United States	mmaasha@broadinstitute.org	FinnGen Teams	**Analysis**
Hannele Laivuori	Institute for Molecular Medicine Finland (FIMM), HiLIFE, University of Helsinki, Helsinki, Finland	hannele.laivuori@helsinki.fi	FinnGen Teams	**Clinical Endpoint Development**
Aki Havulinna	Institute for Molecular Medicine Finland (FIMM), HiLIFE, University of Helsinki, Helsinki, Finland; Finnish Institute for Health and Welfare (THL), Helsinki, Finland	aki.havulinna@helsinki.fi	FinnGen Teams	**Clinical Endpoint Development**
Susanna Lemmelä	Institute for Molecular Medicine Finland (FIMM), HiLIFE, University of Helsinki, Helsinki, Finland	susanna.lemmela@helsinki.fi	FinnGen Teams	**Clinical Endpoint Development**
Tuomo Kiiskinen	Institute for Molecular Medicine Finland (FIMM), HiLIFE, University of Helsinki, Helsinki, Finland	tuomo.kiiskinen@helsinki.fi	FinnGen Teams	**Clinical Endpoint Development**
L. Elisa Lahtela	Institute for Molecular Medicine Finland (FIMM), HiLIFE, University of Helsinki, Helsinki, Finland	laura.lahtela@helsinki.fi	FinnGen Teams	**Clinical Endpoint Development**
Mari Kaunisto	Institute for Molecular Medicine Finland (FIMM), HiLIFE, University of Helsinki, Helsinki, Finland	mari.kaunisto@helsinki.fi	FinnGen Teams	**Communication**
Elina Kilpeläinen	Institute for Molecular Medicine Finland (FIMM), HiLIFE, University of Helsinki, Helsinki, Finland	elina.kilpelainen@helsinki.fi	FinnGen Teams	**E-Science**
Timo P. Sipilä	Institute for Molecular Medicine Finland (FIMM), HiLIFE, University of Helsinki, Helsinki, Finland	timo.p.sipila@helsinki.fi	FinnGen Teams	**E-Science**
Oluwaseun Alexander Dada	Institute for Molecular Medicine Finland (FIMM), HiLIFE, University of Helsinki, Helsinki, Finland	alexander.dada@helsinki.fi	FinnGen Teams	**E-Science**
Awaisa Ghazal	Institute for Molecular Medicine Finland (FIMM), HiLIFE, University of Helsinki, Helsinki, Finland	awaisa.ghazal@helsinki.fi	FinnGen Teams	**E-Science**
Anastasia Kytölä	Institute for Molecular Medicine Finland (FIMM), HiLIFE, University of Helsinki, Helsinki, Finland	anastasia.shcherban@helsinki.fi	FinnGen Teams	**E-Science**
Rigbe Weldatsadik	Institute for Molecular Medicine Finland (FIMM), HiLIFE, University of Helsinki, Helsinki, Finland	rigbe.weldatsadik@helsinki.fi	FinnGen Teams	**E-Science**
Sanni Ruotsalainen	Institute for Molecular Medicine Finland (FIMM), HiLIFE, University of Helsinki, Helsinki, Finland	sanni.ruotsalainen@helsinki.fi	FinnGen Teams	**E-Science**
Kati Donner	Institute for Molecular Medicine Finland (FIMM), HiLIFE, University of Helsinki, Helsinki, Finland	kati.donner@helsinki.fi	FinnGen Teams	**Genotyping**
Timo P. Sipilä	Institute for Molecular Medicine Finland (FIMM), HiLIFE, University of Helsinki, Helsinki, Finland	timo.p.sipila@helsinki.fi	FinnGen Teams	**Genotyping**
Anu Loukola	Helsinki Biobank / Helsinki University and Hospital District of Helsinki and Uusimaa, Helsinki	anu.loukola@hus.fi	FinnGen Teams	**Sample Collection Coordination**
Päivi Laiho	THL Biobank / Finnish Institute for Health and Welfare (THL), Helsinki, Finland	paivi.laiho@thl.fi	FinnGen Teams	**Sample Logistics**
Tuuli Sistonen	THL Biobank / Finnish Institute for Health and Welfare (THL), Helsinki, Finland	tuuli.sistonen@thl.fi	FinnGen Teams	**Sample Logistics**
Essi Kaiharju	THL Biobank / Finnish Institute for Health and Welfare (THL), Helsinki, Finland	essi.kaiharju@thl.fi	FinnGen Teams	**Sample Logistics**
Markku Laukkanen	THL Biobank / Finnish Institute for Health and Welfare (THL), Helsinki, Finland	markku.laukkanen@thl.fi	FinnGen Teams	**Sample Logistics**
Elina Järvensivu	THL Biobank / Finnish Institute for Health and Welfare (THL), Helsinki, Finland	elina.jarvensivu@thl.fi	FinnGen Teams	**Sample Logistics**
Sini Lähteenmäki	THL Biobank / Finnish Institute for Health and Welfare (THL), Helsinki, Finland	sini.lahteenmaki@thl.fi	FinnGen Teams	**Sample Logistics**
Lotta Männikkö	THL Biobank / Finnish Institute for Health and Welfare (THL), Helsinki, Finland	lotta.mannikko@thl.fi	FinnGen Teams	**Sample Logistics**
Regis Wong	THL Biobank / Finnish Institute for Health and Welfare (THL), Helsinki, Finland	regis.wong@thl.fi	FinnGen Teams	**Sample Logistics**
Auli Toivola	THL Biobank / Finnish Institute for Health and Welfare (THL), Helsinki, Finland	auli.toivola@thl.fi	FinnGen Teams	**Sample Logistics**
Minna Brunfeldt	THL Biobank / Finnish Institute for Health and Welfare (THL), Helsinki, Finland	minna.brunfeldt@thl.fi	FinnGen Teams	**Registry Data Operations**
Hannele Mattsson	THL Biobank / Finnish Institute for Health and Welfare (THL), Helsinki, Finland	hannele.mattsson@thl.fi	FinnGen Teams	**Registry Data Operations**
Kati Kristiansson	THL Biobank / Finnish Institute for Health and Welfare (THL), Helsinki, Finland	kati.kristiansson@thl.fi	FinnGen Teams	**Registry Data Operations**
Susanna Lemmelä	Institute for Molecular Medicine Finland (FIMM), HiLIFE, University of Helsinki, Helsinki, Finland	susanna.lemmela@helsinki.fi	FinnGen Teams	**Registry Data Operations**
Sami Koskelainen	THL Biobank / Finnish Institute for Health and Welfare (THL), Helsinki, Finland	sami.koskelainen@thl.fi	FinnGen Teams	**Registry Data Operations**
Tero Hiekkalinna	THL Biobank / Finnish Institute for Health and Welfare (THL), Helsinki, Finland	tero.hiekkalinna@helsinki.fi	FinnGen Teams	**Registry Data Operations**
Teemu Paajanen	THL Biobank / Finnish Institute for Health and Welfare (THL), Helsinki, Finland	teemu.paajanen@thl.fi	FinnGen Teams	**Registry Data Operations**
Priit Palta	Institute for Molecular Medicine Finland (FIMM), HiLIFE, University of Helsinki, Helsinki, Finland	priit.palta@helsinki.fi	FinnGen Teams	**Sequencing Informatics**
Kalle Pärn	Institute for Molecular Medicine Finland (FIMM), HiLIFE, University of Helsinki, Helsinki, Finland	kalle.parn@helsinki.fi	FinnGen Teams	**Sequencing Informatics**
Mart Kals	Institute for Molecular Medicine Finland (FIMM), HiLIFE, University of Helsinki, Helsinki, Finland	mart.kals@helsinki.fi	FinnGen Teams	**Sequencing Informatics**
Shuang Luo	Institute for Molecular Medicine Finland (FIMM), HiLIFE, University of Helsinki, Helsinki, Finland	shuang.luo@helsinki.fi	FinnGen Teams	**Sequencing Informatics**
Tarja Laitinen	Pirkanmaa Hospital District, Tampere, Finland	tarja.laitinen@pshp.fi	FinnGen Teams	**Trajectory**
Mary Pat Reeve	Institute for Molecular Medicine Finland (FIMM), HiLIFE, University of Helsinki, Helsinki, Finland	mary.reeve@helsinki.fi	FinnGen Teams	**Trajectory**
Shanmukha Sampath Padmanabhuni	Institute for Molecular Medicine Finland (FIMM), HiLIFE, University of Helsinki, Helsinki, Finland	sam.padmanabhuni@helsinki.fi	FinnGen Teams	**Trajectory**
Marianna Niemi	University of Tampere, Tampere, Finland	marianna.niemi@tuni.fi	FinnGen Teams	**Trajectory**
Harri Siirtola	University of Tampere, Tampere, Finland	harri.siirtola@tuni.fi	FinnGen Teams	**Trajectory**
Javier Gracia-Tabuenca	University of Tampere, Tampere, Finland	javier.graciatabuenca@tuni.fi	FinnGen Teams	**Trajectory**
Mika Helminen	University of Tampere, Tampere, Finland	mika.helminen@tuni.fi	FinnGen Teams	**Trajectory**
Tiina Luukkaala	University of Tampere, Tampere, Finland	tiina.luukkaala@tuni.fi	FinnGen Teams	**Trajectory**
Iida Vähätalo	University of Tampere, Tampere, Finland	iida.vahatalo@epshp.fi	FinnGen Teams	**Trajectory**
Jyrki Pitkänen	Institute for Molecular Medicine Finland (FIMM), HiLIFE, University of Helsinki, Helsinki, Finland	jyrki.pitkanen@helsinki.fi	FinnGen Teams	**Data protection officer**
Marco Hautalahti	Finnish Biobank Cooperative - FINBB	marco.hautalahti@finbb.fi	FinnGen Teams	**FINBB - Finnish biobank cooperative**
Johanna Mäkelä	Finnish Biobank Cooperative - FINBB	johanna.makela@finbb.fi	FinnGen Teams	**FINBB - Finnish biobank cooperative**
Sarah Smith	Finnish Biobank Cooperative - FINBB	sarah.smith@finbb.fi	FinnGen Teams	**FINBB - Finnish biobank cooperative**
Tom Southerington	Finnish Biobank Cooperative - FINBB	tom.southerington@finbb.fi	FinnGen Teams	**FINBB - Finnish biobank cooperative**
