# Supplementary material for: Mono- and biallelic variant effects on disease at biobank scale
Source: Nature. 2023 Jan 18;613(7944):519–25. doi: 10.1038/s41586-022-05420-7 (PMC9849130; doi:10.1038/s41586-022-05420-7)
Supplement: Supplementary file 2 — Reporting Summary [file 41586_2022_5420_MOESM2_ESM.pdf]

nature research

Corresponding author(s): Henrike O. Heyne, Mark J. Daly

Last updated by author(s): Jul 18, 2022

Reporting Summary

Nature Research wishes to improve the reproducibility of the work that we publish. This form provides structure for consistency and transparency in reporting. For further information on Nature Research policies, see our Editorial Policies and the Editorial Policy Checklist.

Statistics

For all statistical analyses, confirm that the following items are present in the figure legend, table legend, main text, or Methods section.

n/a	Confirmed	
☐	☒	The exact sample size (*n*) for each experimental group/condition, given as a discrete number and unit of measurement
☐	☒	A statement on whether measurements were taken from distinct samples or whether the same sample was measured repeatedly
☐	☒	The statistical test(s) used AND whether they are one- or two-sided<br>*Only common tests should be described solely by name; describe more complex techniques in the Methods section.*
☐	☒	A description of all covariates tested
☐	☒	A description of any assumptions or corrections, such as tests of normality and adjustment for multiple comparisons
☐	☒	A full description of the statistical parameters including central tendency (e.g. means) or other basic estimates (e.g. regression coefficient) AND variation (e.g. standard deviation) or associated estimates of uncertainty (e.g. confidence intervals)
☐	☒	For null hypothesis testing, the test statistic (e.g. F, t, r) with confidence intervals, effect sizes, degrees of freedom and P value noted<br>*Give P values as exact values whenever suitable.*
☐	☒	For Bayesian analysis, information on the choice of priors and Markov chain Monte Carlo settings
☐	☒	For hierarchical and complex designs, identification of the appropriate level for tests and full reporting of outcomes
☐	☒	Estimates of effect sizes (e.g. Cohen's *d*, Pearson's *r*), indicating how they were calculated

Our web collection on statistics for biologists contains articles on many of the points above.

Software and code

Policy information about availability of computer code

Data collection	Genotype calls were made with GenCall and zCall algorithms for Illumina and AxiomGT1 algorithm for Affymetrix data. Genotype imputation was done with Beagle 4.1 (version 08Jun17.d8b) with the population-specific SISu v3 reference panel created from high-quality WGS data of 3,775 individuals. Further processing of the genotype data was done using standard genome analysis software Plink 1.9 and 2.0 and BCFtools 1.7 and 1.9.
Data analysis	We used the SAIGE (version 0.35.8.8) software for running all R4 GWAS with additive and recessive models. Pipelines for parallel computing were created using Cromwell-29 and 31 and Wdltool-0.14. Fine-mapping was done with SuSiE. Further processing of the data was done using R 3.4.1 (packages: data.table 1.10.4, sm 2.2-5.4). Statistical analyses and figures were done using additional R packages ggplot2, plyr, survminer, survival, tidyr, Rutils.

For manuscripts utilizing custom algorithms or software that are central to the research but not yet described in published literature, software must be made available to editors and reviewers. We strongly encourage code deposition in a community repository (e.g. GitHub). See the Nature Research guidelines for submitting code & software for further information.

Data

Policy information about availability of data

All manuscripts must include a data availability statement. This statement should provide the following information, where applicable:

- Accession codes, unique identifiers, or web links for publicly available datasets
- A list of figures that have associated raw data
- A description of any restrictions on data availability

All summary statistics described in this manuscript can be found in the Supplementary Tables. A full list of FinnGen endpoints for release 4 is available at https://www.finngen.fi/en/researchers/clinical-endpoints.

Field-specific reporting

Please select the one below that is the best fit for your research. If you are not sure, read the appropriate sections before making your selection.

☒ Life sciences ☐ Behavioural & social sciences ☐ Ecological, evolutionary & environmental sciences

For a reference copy of the document with all sections, see nature.com/documents/nr-reporting-summary-flat.pdf

Life sciences study design

All studies must disclose on these points even when the disclosure is negative.

Sample size	All 176,899 participants in FinnGen datafreeze 4 were included in the study.
Data exclusions	In FinnGen datafreeze 4, individuals with missing minimum phenotype data or a mismatch between imputed sex and sex in registry data as well as population outliers in PCA have been excluded.
Replication	All homozygous associations we present in Table 1 including the novel hits we highlight in the manuscript and in a Supplementary Note were validated in FinnGen datafreeze 6 (n=234,553) and/or in the UK biobank (n=420,531).
Randomization	GWAS were performed in individuals with versus without one of 2,444 disease endpoints. In all GWAS age, sex, genotyping batch and the first ten PCs of genotypes were included as covariates.
Blinding	In hypothesis free GWAS, blinding is not possible/necessary.

Reporting for specific materials, systems and methods

We require information from authors about some types of materials, experimental systems and methods used in many studies. Here, indicate whether each material, system or method listed is relevant to your study. If you are not sure if a list item applies to your research, read the appropriate section before selecting a response.

Materials & experimental systems

n/a	Involved in the study	
☒	☐	Antibodies
☒	☐	Eukaryotic cell lines
☒	☐	Palaeontology and archaeology
☒	☐	Animals and other organisms
☐	☒	Human research participants
☐	☒	Clinical data
☒	☐	Dual use research of concern

Methods

n/a	Involved in the study	
☒	☐	ChIP-seq
☒	☐	Flow cytometry
☒	☐	MRI-based neuroimaging

Human research participants

Policy information about studies involving human research participants

Population characteristics	Participants are on average 59.4 ± 17 (mean ± standard deviation) years old, 100,361 are female, 76,538 male. As samples were mainly collected through legacy collections and hospital biobanks the estimated FinnGen participant may be expected to have more diseases than Finnish population average. Phenotype data comes from digital health record data from Finnish health registries.
Recruitment	The collected samples consist of two entities: 1) legacy samples, mainly collected by the THL (National Institute for Health and Welfare in Finland) and 2) prospective samples which were mainly be collected by hospital biobanks. Almost all of the Finnish biobanks are part of the FinnGen study.
Ethics oversight	The FinnGen project has been approved by the Coordinating Ethics Committee of the Helsinki and Uusimaa Hospital District.

Note that full information on the approval of the study protocol must also be provided in the manuscript.

Clinical data

Policy information about clinical studies

All manuscripts should comply with the ICMJE guidelines for publication of clinical research and a completed CONSORT checklist must be included with all submissions.

Clinical trial registration	*Provide the trial registration number from ClinicalTrials.gov or an equivalent agency.*

Study protocol | *Note where the full trial protocol can be accessed OR if not available, explain why.*

Data collection | *Describe the settings and locales of data collection, noting the time periods of recruitment and data collection.*

Outcomes | *Describe how you pre-defined primary and secondary outcome measures and how you assessed these measures.*
